# Kawasaki Disease-Specific Molecules in the Sera Are Linked to Microbe-Associated Molecular Patterns in the Biofilms

**DOI:** 10.1371/journal.pone.0113054

**Published:** 2014-11-20

**Authors:** Takeshi Kusuda, Yasutaka Nakashima, Kenji Murata, Shunsuke Kanno, Hisanori Nishio, Mitsumasa Saito, Tamami Tanaka, Kenichiro Yamamura, Yasunari Sakai, Hidetoshi Takada, Tomofumi Miyamoto, Yumi Mizuno, Kazunobu Ouchi, Kenji Waki, Toshiro Hara

**Affiliations:** 1 Department of Pediatrics, Graduate School of Medical Sciences, Kyushu University, Fukuoka, Japan; 2 Department of Bacteriology, Graduate School of Medical Sciences, Kyushu University, Fukuoka, Japan; 3 Graduate School of Pharmaceutical Sciences, Kyushu University, Fukuoka, Japan; 4 Department of Pediatric Infectious Disease, Fukuoka Children's Hospital and Medical Center for Infectious Disease, Fukuoka, Japan; 5 Department of Pediatrics, Kawasaki Medical School Hospital, Okayama, Japan; 6 Department of Pediatrics, Kurashiki Central Hospital, Okayama, Japan; Loyola University Medical Center, United States of America

## Abstract

**Background:**

Kawasaki disease (KD) is a systemic vasculitis of unknown etiology. The innate immune system is involved in its pathophysiology at the acute phase. We have recently established a novel murine model of KD coronary arteritis by oral administration of a synthetic microbe-associated molecular pattern (MAMP). On the hypothesis that specific MAMPs exist in KD sera, we have searched them to identify KD-specific molecules and to assess the pathogenesis.

**Methods:**

We performed liquid chromatography-mass spectrometry (LC-MS) analysis of fractionated serum samples from 117 patients with KD and 106 controls. Microbiological and LC-MS evaluation of biofilm samples were also performed.

**Results:**

KD samples elicited proinflammatory cytokine responses from human coronary artery endothelial cells (HCAECs). By LC-MS analysis of KD serum samples collected at 3 different periods, we detected a variety of KD-specific molecules in the lipophilic fractions that showed distinct m/z and MS/MS fragmentation patterns in each cluster. Serum KD-specific molecules showed m/z and MS/MS fragmentation patterns almost identical to those of MAMPs obtained from the biofilms formed *in vitro* (common MAMPs from *Bacillus cereus*, *Yersinia pseudotuberculosis* and *Staphylococcus aureus*) at the 1^st^ study period, and from the biofilms formed *in vivo* (common MAMPs from *Bacillus cereus*, *Bacillus subtilis/Bacillus cereus/Yersinia pseudotuberculosis* and *Staphylococcus aureus*) at the 2^nd^ and 3^rd^ periods. The biofilm extracts from *Bacillus cereus*, *Bacillus subtilis*, *Yersinia pseudotuberculosis* and *Staphylococcus aureus* also induced proinflammatory cytokines by HCAECs. By the experiments with IgG affinity chromatography, some of these serum KD-specific molecules bound to IgG.

**Conclusions:**

We herein conclude that serum KD-specific molecules were mostly derived from biofilms and possessed molecular structures common to MAMPs from *Bacillus cereus, Bacillus subtilis*, *Yersinia pseudotuberculosis and Staphylococcus aureus*. Discovery of these KD-specific molecules might offer novel insight into the diagnosis and management of KD as well as its pathogenesis.

## Introduction

The etiology of Kawasaki disease (KD) remains unknown, however, KD has long been considered to be caused by an infectious agent, because of its characteristics of the symptoms, age distribution, seasonality, occurrence of community outbreaks and epidemic cycles. On the other hand, no consistently recoverable agents, lack of person-to-person transmission or a common contagious source, and paucity of case clusters in families, schools or nurseries are supportive of a non-infectious cause for KD [1]–[3]. Temporal clustering and marked seasonality in KD occurrence in Japan, Hawaii and San Diego also suggest a wind-borne environmental trigger for this disease [4].

KD is also characterized by marked elevations of serum levels of proinflammatory cytokines and chemokines [2] and the activation of the innate immune system [5]–[7]. We have established a novel murine model of KD coronary arteritis by oral administration of FK565, which functions as a synthetic microbe-associated molecular pattern (MAMP) and a ligand to one of the innate immune receptors, nucleotide-binding oligomerization domain-containing protein (NOD) 1 [8]. In this report, we performed liquid chromatography-mass spectrometry (LC-MS) analysis of KD sera to find out KD-specific molecules and demonstrated that serum KD-specific molecules were closely linked to MAMPs in the biofilms.

## Materials and Methods

### Study subjects

All patients enrolled in this study were admitted to Kyushu University Hospital, Fukuoka Children's Hospital and Medical Center for Infectious Diseases, Kawasaki Medical School Hospital or Kurashiki Central Hospital between June 2010 and March 2014. The study subjects consisted of 117 patients with KD (median age, 21 months; range 3–96 months; male/female, 65/52), 101 controls with other febrile illnesses (DC: median age, 16 months; range 0–121 months; male/female, 61/40), and 5 normal controls (NC: median age, 6 months; range 3–39 months; male/female, 1/4). A diagnosis of KD was made according to the Diagnostic Guidelines of KD [9]. The Ethical Committee of Kyushu University approved the study. Written informed consent was obtained from all guardians. The 1^st^ study subjects (samples were collected mostly between July 2011 and February 2012) consisted of 43 patients with KD, 41 controls with DC (respiratory syncytial virus infection: *n* = 4, influenza A virus infection: *n* = 7, adenovirus infection: *n* = 2, exanthema subitum: *n* = 5, varicella: *n* = 2, bacteremia: *n* = 2, pneumonia: *n* = 6, tonsillitis: *n* = 1, lymphadenitis: *n* = 5, cellulitis: *n* = 1, urinary tract infection: *n* = 1, gastritis: *n* = 5), and 5 NC. The 2^nd^ (mostly between May 2012 and July 2013) and 3^rd^ (mostly between November 2013 and March 2014) study subjects consisted of 41 KD patients and 30 DC controls (respiratory syncytial virus infection: *n* = 8, influenza A virus infection: *n* = 4, adenovirus infection: *n* = 2, exanthema subitum: *n* = 2, herpetic stomatitis: *n* = 1, pneumonia: *n* = 5, bronchitis: *n* = 2, upper respiratory infection: *n* = 1, tonsillitis: *n* = 2, deep neck abscess: *n* = 1, acute otitis media: *n* = 1, urinary tract infection: *n* = 1), and 33 KD patients and 30 DC controls (respiratory syncytial virus infection: *n* = 8, influenza A virus infection: *n* = 1, adenovirus infection: *n* = 1, pneumonia: *n* = 6, bronchitis: *n* = 1, upper respiratory infection: *n* = 4, tonsillitis: *n* = 2, lymphadenitis: *n* = 1, sinusitis: *n* = 1, acute otitis media: *n* = 1, urinary tract infection: *n* = 2, gastritis: *n* = 2), respectively.

### Sample collection

Blood samples were collected at the time of routine examinations before and after high-dose intravenous immunoglobulin (IVIG) therapy, and after resolution of symptoms. The sera were separated by centrifugation and stored at −30°C until the analysis. Routine bacterial cultures were performed with throat, tongue, nasal and rectal swabs. Biofilms from teeth, tongue, nasal cavity, or rectum (stool) were collected by cotton swabs or interdental brushes (for teeth). These swabs or brushes were suspended in double distilled water (ddH_2_O) immediately and stored at −30°C until the analysis. Simultaneous collection of biofilm and serum samples was performed at 2^nd^ (*n* = 12, mostly October-December, 2012) and 3^rd^ (*n* = 11, mostly January-February, 2014) study periods.

### Lipid extraction

Serum samples or other specimens were separated into lipophilic and hydrophilic fractions by Folch method [10] or ethyl acetate extraction [11], [12]. As for Folch method [10], 100 µL of serum was acidified to pH5 with acetic acid and mixed with 2∶1 chloroform-methanol mixture (v/v) to a final volume 300 µL. The mixture was shaken and centrifuged at 3000 rpm for 10 minutes, and the bottom lipophilic layer and upper hydrophilic layer were collected and evaporated. The lipophilic pellet was dissolved in 5 µL of chloroform, 5 µL of dimethyl sulfoxide (DMSO), and 40 µL of ddH_2_O and hydrophilic pellet was in 50 µL of ddH_2_O. As for ethyl acetate extraction [11], [12], 100 µL of serum was mixed with the same volume of ethyl acetate. After centrifugation, the upper lipophilic layer including the interface and the bottom hydrophilic layer were transferred, evaporated, and dissolved in 50 µL of 20% methanol (lipophilic layers), and in 100 µL ddH_2_O (hydrophilic layers) for cell stimulation, respectively. Since the human coronary artery endothelial cell (HCAEC)-stimulatory activities of KD serum samples were not stable after extraction with Folch method, we used ethyl acetate instead of chloroform. For LC-MS, lipophilic fractions were dissolved in 100% methanol. Other samples were also mixed with the same volumes of ethyl acetate, and centrifuged. Upper lipophilic layers including interfaces were collected, evaporated and dissolved in 100% methanol. To each sample, dibutyl hydroxytoluene was added at a final concentration of 1.0% as an antioxidant [13].

### Cell stimulation

HCAECs (purchased from Lonza and no mycoplasma contamination) were cultured in EBM-2 medium with EGM-2MV (Lonza) in a 5% CO_2_ incubator at 37°C. These cells, between passages 5 and 7, were suspended and seeded into 75 cm^2^ flask. After passage, HCAECs were introduced in a 96 well plate (3×10^3^ cells/well). On the following day, the medium was changed and the supernatants were collected for assay 24 hours after stimulation.

### Cytokine assay

The concentrations of IL-8, IL-6, IL-1β, TNF-α, IL-12p70, and IL-10 in culture supernatants were measured by EC800 cell analyzer (Sony Corporation) with a BD Cytometric Bead Array human inflammation kit (BD Biosciences) [8]. We performed the experiments at least 3 times.

### LC- MS analysis

Samples were analyzed by high performance liquid chromatography (HPLC, Agilent 1200 HPLC instrument, Agilent Technologies) on Dionex Acclaim surfactant column (3 µm, 120Å, 2.1×150 mm, DIONEX) and MS (Esquire 6000 electrospray ionization: ESI, Bruker Daltonics). The mobile phases were H_2_O with 0.1% formic acid (eluent A) and acetonitril with 0.1% formic acid (eluent B). They were delivered at a flow rate of 0.2 ml/min and the column was operated at 25°C. The gradient was as follows: 0–3 min. 20% B, 3–12 min. 20–100% B, 12–70 min. 100% B. The injection volume to the system was fixed at 10 µl. The column eluent was connected to MS. The ESI-MS^n^ spectrum conditions were optimized in the negative-ion mode with the conditions as follows: nebulizer gas, 30.0 psi; drying gas, flow 8 l/min; dry temperature 330°C; high voltage (HV) capillary, 4500 V; HV end plate offset, −500 V; target ion trap, 30000; scan range 100–3000 m/z. The width for targeted precursor ions was set at 4 m/z.

### Biofilm extraction from glass slides

After removing the medium, the culture tube and glass slides were washed once with PBS and vortexed in the presence of ethyl acetate. The ethyl acetate was transferred and evaporated, and the pellet was dissolved in 100% methanol. Details were described in Text S1 in File S1.

### IgG affinity chromatography

Columns used included human polyclonal IgG-conjugated Sepharose 6 Fast (GE Healthcare Life Science), human IgG F(ab′)_2_ fragment-conjugated agarose (ROCKLAND), human IgG Fc fragment full length protein (Abcam)-coupled to cyanogen-bromide (CNBr) Sepharose 4B (GE Healthcare Life Science), mouse monoclonal IgG against a specific antigen (Myc-tag)-conjugated agarose (MBL), rabbit monoclonal IgG against a specific antigen (Phospho-Met (Tyr1234/1235) (D26) XP)-conjugated sepharose (Cell Signaling), and inactivated CNBr Sepharose 4B (GE Healthcare Life Science). Coupling to and inactivation of CNBr Sepharose 4B were performed according to the manufacturer's instructions. Each column was washed once with 10 volumes of PBS with 0.05% Tween20, and twice with 20 volumes of PBS. Biofilms extracts dissolved in PBS with 20% methanol or sera without dilution were applied to a column. After incubation for 30–60 minutes, the mixture was centrifuged and washed twice with PBS. Elution was performed with ethyl acetate. The ethyl acetate elutes were evaporated and the pellets were dissolved in 100% methanol. Inactivated CNBr Sepharose 4B was used as a control column. We performed the experiments at least 3 times.

### Statistics

Data were analyzed by Welch's *t*-test and Fisher's exact test using a statistical software, JMP version 8.0 (SAS Institute), and *P*-values of <0.05 were considered to be statistically significant.

## Results

### Activation of HCAECs by KD sera *in vitro*

**Figure 1:**
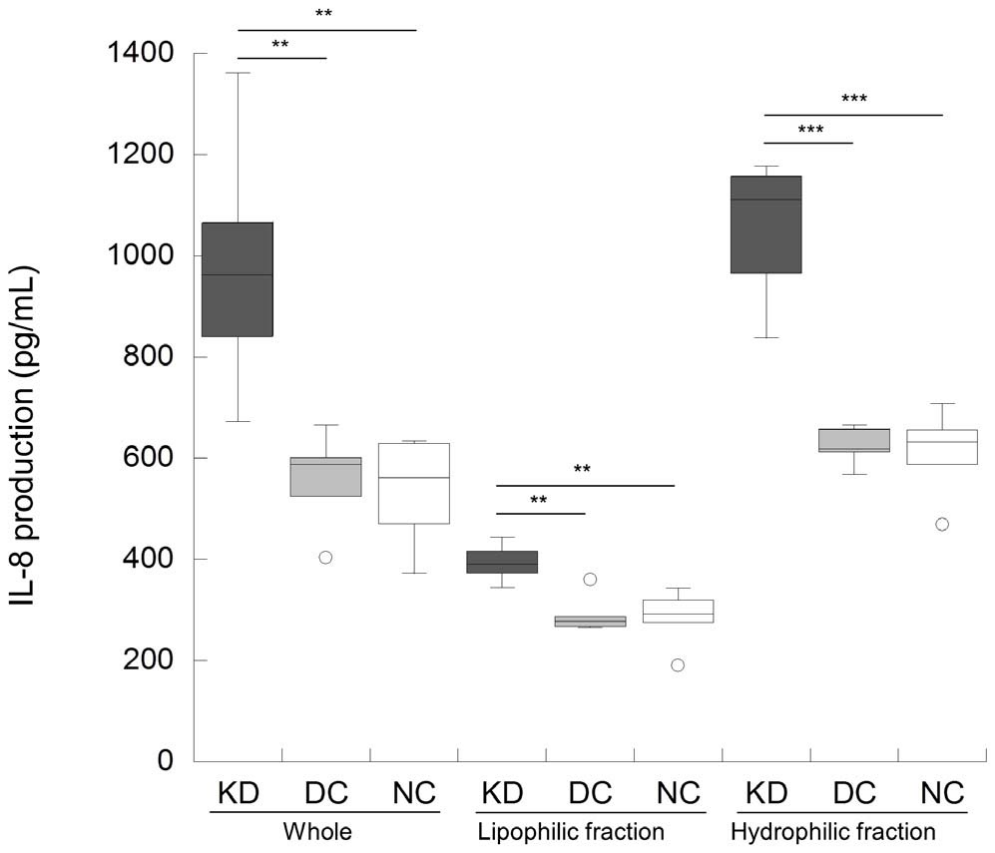
Whole and fractionated serum samples from KD patients induce cytokine production in HCAECs. The production of IL-8 by HCAECs was measured in triplicate after 24-hour stimulation with whole sera or lipophilic and hydrophilic fractions from KD patients (n = 6), DC controls (n = 5; pneumonia; n = 2, influenza A virus infection; n = 1, adenovirus infection; n = 1 and urinary tract infection; n = 1), or NC subjects (n = 5). Lipophilic and hydrophilic fractions were separated by ethyl acetate extraction. The bottom and top edges of the box plot correspond to the 25th and 75th percentiles, respectively. The horizontal line inside the box represents the median of the distribution. The whiskers indicate the 10th and 90th percentiles. **P<0.01; ***P<0.001 (Welch's *t*-test).

Since NOD1 ligand directly activates endothelial cells [8] and the expression of endothelial activation antigens was detected in KD skin biopsy specimens [14], HCAECs were employed for the search of such molecules as MAMPs in KD sera. KD samples induced significantly higher IL-8 production than DC and NC samples in whole sera. After separation into the lipophilic and hydrophilic fractions with ethyl acetate, KD samples elicited higher IL-8 production in each fraction (Figure 1). Similar results were obtained regarding IL-6 production. IL-6 and IL-8 levels in most of the tested sera from KD patients were under detection limits or negligible (data not shown). These results suggested that sera from KD patients contained molecules that stimulated HCAECs to produce IL-8 and IL-6. NOD1-stimulatory activity was also examined in whole and fractionated serum samples from KD, DC and NC, as described in Text S1 in File S1. However, no NOD1 activity was detected in any of these samples (data not shown).

### Serum KD-specific molecules common to MAMPs from the *in vitro* biofilms

**Figure 2:**
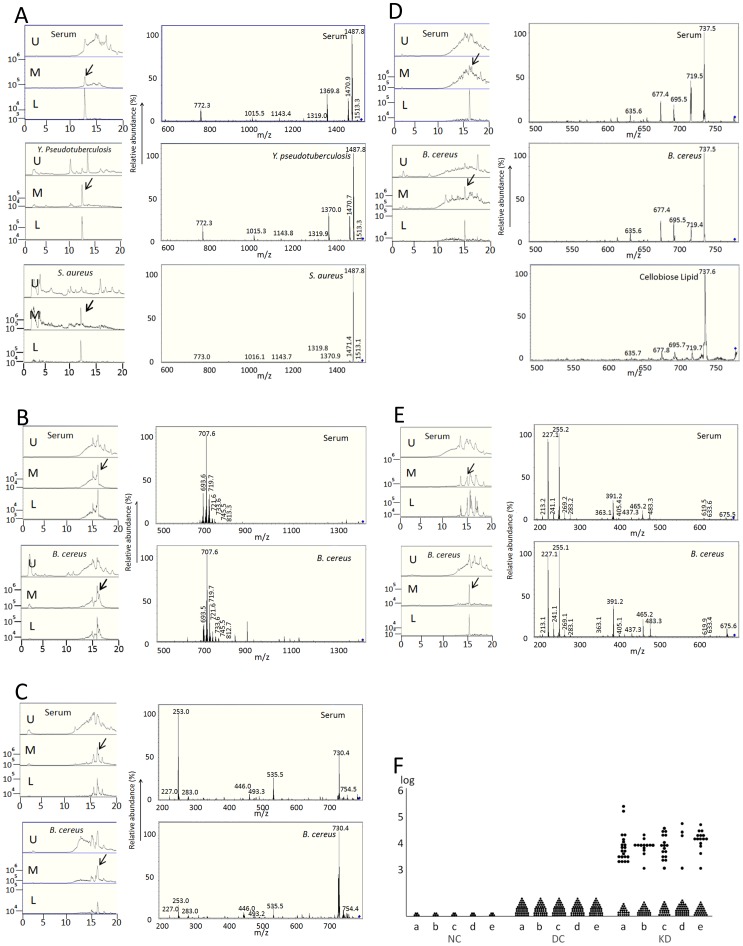
LC-MS chromatograms and MS/MS fragmentation patterns of serum KD-specific molecules at the 1^st^ study period. A–E: Each left upper panel: LC-MS chromatograms of KD-specific molecules (A: m/z 1531.8, B: m/z 1414.3, C: m/z 790.9, D: m/z 779.8, and E: m/z 695.0), Each left lower panel: LC-MS chromatograms of biofilm extracts (or initial culture supernatants) from *Y. pseudotuberculosis* and *S. aureus* (A) and *B. cereus* (B–E). U: Total ion current chromatograms, M: Extracted-ion chromatograms at m/z 1500–1600 (A), m/z 1400–1500 (B), m/z 700–800 (C and D), and m/z 600–700 (E), L: Extracted-ion chromatograms at m/z 1531.8 (A), m/z 1414.3 (B), m/z 790.9 (C), m/z 779.8 (D), and m/z 695.0 (E). Arrows indicate peaks of target molecules. Each right upper panel: MS/MS fragmentation patterns of KD-specific molecules (A: m/z 1531.8, B: m/z 1414.3, C: m/z 790.9, D: m/z 779.8, and E: m/z 695.0), Each right lower panel: MS/MS fragmentation patterns of biofilm extracts (or initial culture supernatants) from *Y. pseudotuberculosis* and *S. aureus* (A) and *B. cereus* (B–E). As for the molecule at m/z 779.8, cellobiose lipid shows a MS/MS fragmentation pattern similar to that of KD sera (D, right lowest panel). The intensity is shown by relative abundance. F: The detection rates of each molecule in NC (N = 5), DC (N = 41) or KD (N = 43) sera are shown. Twenty-one (48.8%) of 43 are positive at m/z 1531.8 (a), 13 (30.2%) of 43 at m/z 1414.3 (b), 17 (39.5%) of 43 at m/z 790.9 (c), 4 (9.3%) of 43 at m/z 779.8 (d) and 15 (34.9%) of 43 at m/z 695.0 (e) when the intensity above 1×10^3^ is considered to be significant. The overall detection rate was 76.7% (33 of 43). *P*<0.0001 (a, b, c and e); *P* = 0.0364 (d) (Fisher's exact test).

We explored serum KD-specific molecules in the lipophilic and hydrophilic fractions by LC-MS analysis, and found numerous KD-specific molecules in the lipophilic fractions in 10 KD patients of the 1^st^ study period (data not shown). It has been reported that *Yersinia (Y.) pseudotuberculosis*-infected children sometimes develop KD [15], [16]. Moreover, *Bacillus (B.) cereus* and *B. subtilis* were 2 major spore-forming bacteria isolated from KD patients (Table S1 in File S1), which might work as possible wind-borne environmental triggers for KD [4]. Therefore, to find out the MAMPs identical to serum KD-specific molecules, we initially analyzed culture supernatants (later biofilms) of *Y. pseudotuberculosis, B. cereus* and *B. subtilis* from KD patients by LC-MS. Five KD-specific molecules at m/z 1531.8, 1414.3, 790.9, 779.8, and 695.0 showed the m/z and MS/MS fragmentation patterns almost identical to those of the MAMPs from *Y. pseudotuberculosis* and *B. cereus* (Figure 2 and Figure S1 in File S1). The 5 serum KD-specific molecules were detected with 100% specificity and 9.3%–48.8% sensitivity. At least one of the 5 KD-specific molecules was detected in 33 (76.7%) out of 43 patients at the 1^st^ study period (Figure 2, Table 1). All serum KD-specific molecules decreased after IVIG treatment (Figure S1F in File S1). By comparison with 5 authentic microbial glycolipids, only one molecule at m/z 779.8 showed a MS/MS fragmentation pattern similar to that of cellobiose lipid (Figure 2D).

**Table 1:** The detection rates of serum KD-specific MAMPs at each study.

	1^st^ KD study serum MAMPs						2^nd^ KD study serum MAMPs					3^rd^ KD study serum MAMPs		
	1531.8	1414.3	790.9	779.8	695.0		1171.4	1169.4	906.8	695.0		667.4	619.4	409.3
	% positive (positive number)						% positive (positive number)					% positive (positive number)		
1^st^ KD study *n* = 43	48.8 (21)	30.2 (13)	39.5 (17)	9.3 (4)	34.9 (15)		0.0 (0)	0.0 (0)	0.0 (0)	34.9 (15)		0.0 (0)	4.7 (2)	2.3 (1)
	76.7^*^ (33)						34.9^*^ (15)					7.0^*^ (3)		
2^nd^ KD study n = 41						Pre	0.0 (0)	0.0 (0)	0.0 (0)	0.0 (0)				
	0.0 (0)	0.0 (0)	5.4 (2)	0.0 (0)	16.2 (6)	SBA period	16.7 (2)	16.7 (2)	33.3 (4)	50.0 (6)		0.0 (0)	0.0 (0)	0.0 (0)
						Post	0.0 (0)	0.0 (0)	10.0 (1)	0.0 (0)				
			16.2^*^ (6)				SBA period: 83.3^*^ (10)					0.0^*^ (0)		
							Pre & Post: 3.4^*^ (1)							
3^rd^ KD study n = 33											Pre	0.0 (0)	0.0 (0)	0.0 (0)
	0.0 (0)	0.0 (0)	0.0 (0)	0.0 (0)	0.0 (0)		0.0 (0)	0.0 (0)	16.1 (5)	0.0 (0)	SBA period	63.6 (7)	45.5 (5)	81.8 (9)
											Post	0.0 (0)	0.0 (0)	0.0 (0)
	0.0^*^ (0)						16.1^*^ (5)					SBA period: 90.9^*^ (10)		
												Pre & Post: 0.0^*^ (0)		

At the 2^nd^ and 3^rd^ studies, both *in vivo* biofilms and serum samples were simultaneously collected. *In vivo* biofilms samples were searched for MAMPs common to those in serum samples by LC-MS and MS/MS analyses. SBA: simultaneous biofilm analysis, *: overall % positive (overall positive numbers). DC samples at the 1^st^ (*n* = 41), 2^nd^ (*n* = 30) and 3^rd^ (*n* = 30) study periods were all negative for all KD MAMPs. The detection rates of KD-specific serum MAMPs between KD samples (*n* = 43) and DC samples (*n* = 41) at the 1^st^ study showed statistically significant differences at m/z 1531.8, m/z 1414.3, m/z 790.9, m/z 695.0, and overall (*P*<0.0001), but not at m/z 779.8 (*P* = 0.1164) by Fisher's exact test. The detection rates between SBA period KD samples (*n* = 12) and DC samples (*n* = 30) at the 2^nd^ study showed statistically significant differences at m/z 906.8 (*P* = 0.0044), m/z 695.0 (*P* = 0.0002) and overall (*P*<0.0001), but not at m/z 1171.4 (*P* = 0.0767) and m/z 1169.4 (*P* = 0.0767). The detection rates between SBA period KD samples (*n* = 11) and DC samples (*n* = 30) at the 3^rd^ study showed statistically significant differences at all 3 molecules and overall (*P*<0.0001).

As these microbes ceased production of these MAMPs after 1 or 2 passages, we investigated the optimal culture conditions (medium, temperature, duration, shaking, nutrition and biofilm formation) for the production of these MAMPs. We found that they produced these MAMPs reproducibly in the biofilm-forming conditions in the presence of lipid, especially butter (Figure S2 in File S1). We thus examined the culture supernatants and biofilm extracts from all the spore-forming microbes isolated from KD patients as well as additional microbes by LC-MS and MS/MS analyses. In addition to the 3 bacteria mentioned above, almost all KD-specific molecules were detected not in the culture supernatants but in the biofilm extracts. Although a KD-specific molecule at m/z 1531.8 was detected in biofilm extracts from several bacteria (Table S2 in File S1), *Y. pseudotuberculosis* and *Staphylococcus (S.) aureus* were isolated from KD patients. In addition, *B. cereus*-associated MAMPs were detected in the sera of KD patients from whom *B. cereus* was actually isolated (Figure 2, Figure S1 in File S1 and Table S1 in File S1).

### Serum KD-specific molecules common to MAMPs from the *in vivo* biofilms

**Figure 3:**
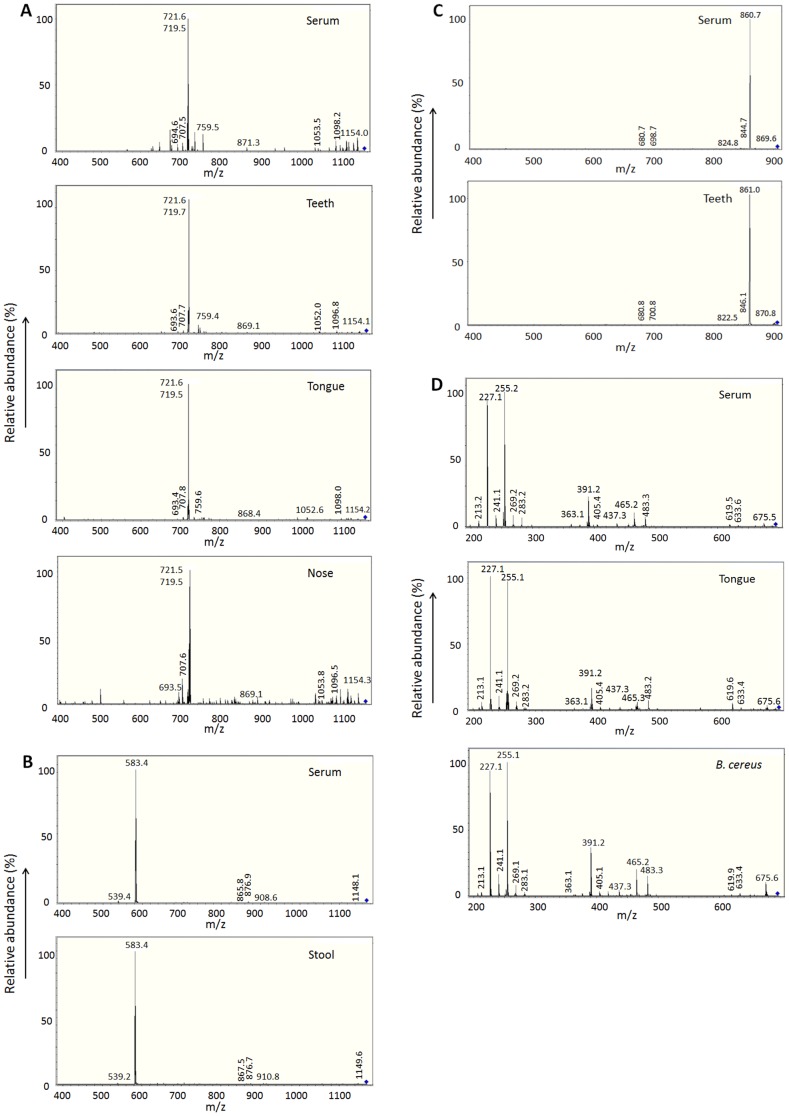
KD *in vivo* biofilms contain MAMPs common to serum KD-specific molecules (2^nd^ study period). Extensive search for common molecules in the *in vivo* biofilms and sera from KD patients or DC controls revealed that 4 KD-specific molecules (m/z 1171.4, 1169.4, 906.8, and 695.0) showed similar MS/MS fragmentation patterns between the two in KD patients (Table S3 in File S1). A: The molecule at m/z 1171.4 was common in KD serum and biofilm extracts from teeth, tongue, or nose. B: The molecule at m/z 1169.4 was common in KD serum and stool biofilm extracts. C: The molecule at m/z 906.8 was common in KD serum and teeth biofilm extracts. D: The molecule at m/z 695.0 was common in KD serum and tongue biofilm extracts and *in vitro* biofilm extracts from *B. cereus*.

Although numerous KD-specific molecules were present in the lipid extracts from KD serum samples of the 2^nd^ study period, the 5 KD-specific MAMPs observed at the 1^st^ study period were no longer detected in the tested 10 samples. As the number of oligosaccharides, and the length, position, degree of saturation and configuration of the hydrophobic moieties in microbial glycolipids are known to change according to the environmental conditions and microbial origins [17], [18], we examined lipid extracts from the *in vivo* biofilms in respective KD patients by LC-MS analysis. We detected 4 serum KD-specific molecules with MS/MS fragmentation patterns similar to one (m/z 695.0) of the 5 MAMPs at the 1^st^ study period and 3 additional ones in the biofilms formed *in vivo* (teeth, tongue, nose and stool), respectively, in 10 (83.3%) out of 12 KD patients (Table S3 in File S1, Figure 3, Table 1). By the analysis of 20 microbial biofilm extracts and 5 authentic glycolipids, only one molecule at m/z 695.0 in tongue biofilms showed a MS/MS fragmentation pattern similar to that of a MAMP of *B. cereus* (Table S2 in File S1).

**Figure 4:**
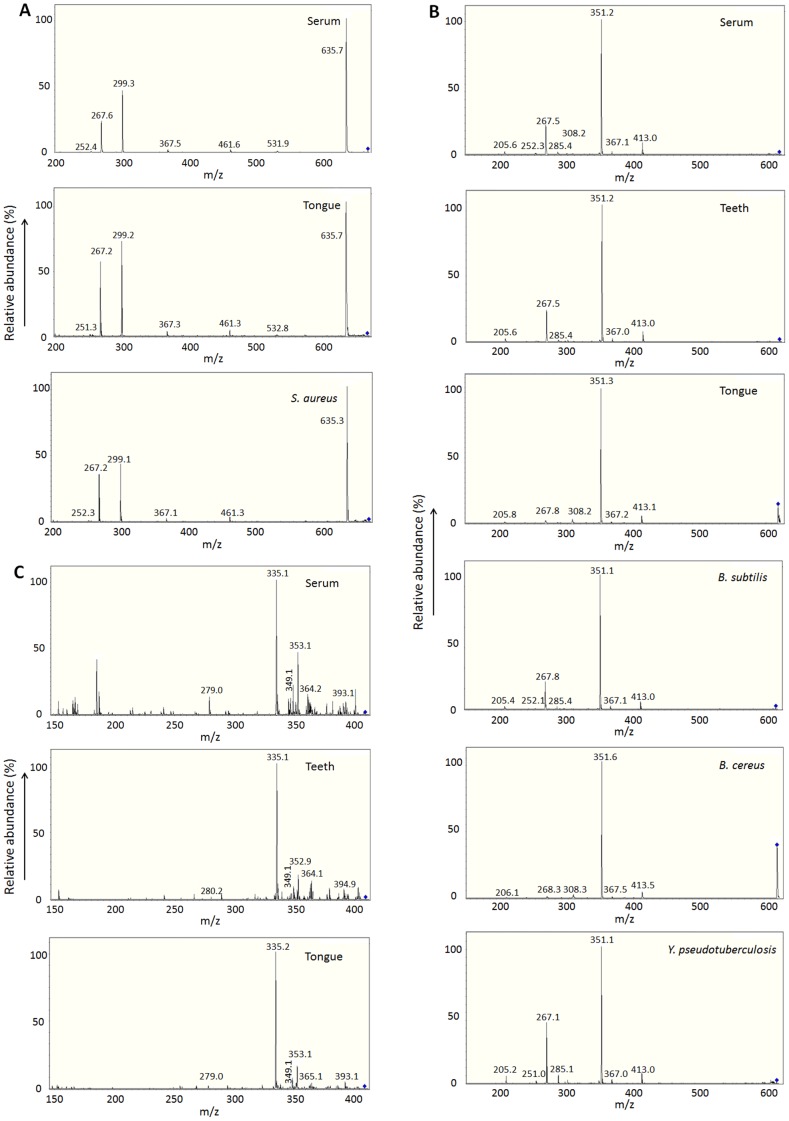
KD *in vivo* biofilms contain MAMPs common to serum KD-specific molecules (3^rd^ study period). Three serum KD-specific molecules (m/z 667.4, 619.4 and 409.3) at the 3^rd^ study showed the same m/z with MS/MS fragmentation patterns similar to MAMPs from *in vivo* biofilm extracts (Table S4 in File S1) and *in vitro* bacterial biofilm extracts. A: The molecule at m/z 667.4 was common in KD serum, tongue biofilm extracts and *in vitro* biofilm extracts from *S. aureus*. B: The molecule at m/z 619.4 was common in KD serum, teeth and tongue biofilm extracts, and *in vitro* biofilm extracts from *B. subtilis*, *B. cereus* and *Y. pseudotuberculosis*. C: The molecule at m/z 409.3 was common in KD serum, and teeth and tongue biofilm extracts.

At the 3^rd^ study period, we examined teeth and tongue biofilms and found 3 distinct KD-specific molecules with MS/MS fragmentation patterns similar to those from the *in vivo* biofilms in the respective KD patients by LC-MS and MS/MS analyses (Table S4 in File S1, Figure 4). Two of the 3 serum KD-specific molecules showed the MS/MS fragmentation patterns similar to a MAMP from *S. aureus*, and that from *B. subtilis*, *B. cereus* or *Y. pseudotuberculosis*, respectively. Actually, *B. subtilis* and *S. aureus* were detected from the patients. At least one of the 3 KD-specific MAMPs was detected in 10 (90.9%) out of 11 KD patients. The detection rates of KD-specific serum MAMPs at the 1^st^, 2^nd^ and 3^rd^ study periods are shown in Table 1. By LC-MS analysis, all the 106 control samples were negative for the 5, 4 and 3 KD-specific MAMPs detected at the 1^st^, 2^nd^ and 3^rd^ study periods, respectively.

### IgG sepharose binds some serum KD-specific MAMPs

**Figure 5:**
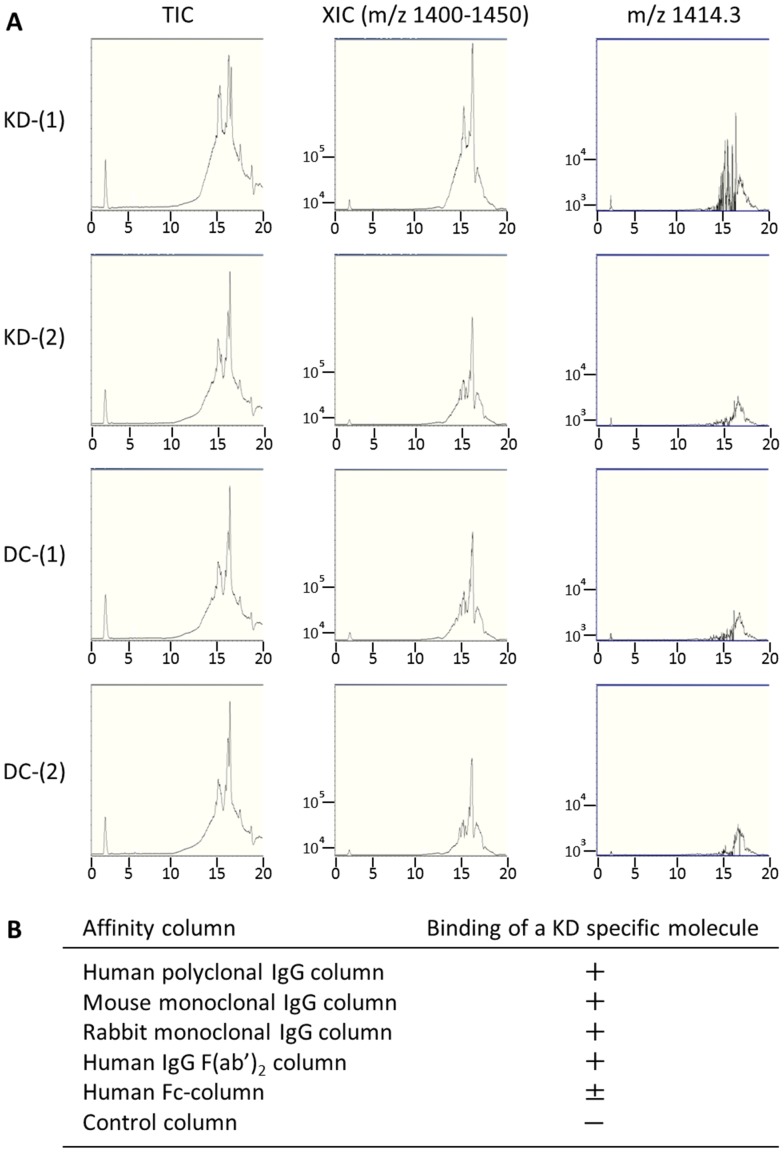
LC-MS chromatograms of IgG sepharose-binding molecules. A. Representative LC-MS chromatograms of a IgG sepharose-binding molecule (m/z 1414.3) are shown in a KD patient and a DC control. TIC: Total ion current chromatograms, XIC: Extracted-ion chromatograms at m/z 1400–1500, and extracted-ion chromatograms at m/z 1414.3. (1) Human polyclonal IgG-conjugated sepharose 6 Fast (2) Inactivated CNBr Sepharose 4B control column. B. Binding of a KD-specific molecule to various affinity columns: Columns used are described in ONLINE METHODS. +: The binding quantities of a KD-specific molecule analyzed by LC-MS were equal or larger than those to human polyclonal IgG column, ±: smaller than 20% of those to human polyclonal IgG column, -: no binding. We performed the experiments 3 times.

It has been reported that certain microbial glycolipids bound to various species of IgG [19], [20]. Therefore, we checked IgG-binding activity of KD-specific MAMPs using various kinds of IgG affinity columns. LC-MS analysis of IgG sepharose-binding molecules were conducted on KD and DC samples. Three KD-specific IgG sepharose-binding molecules were detected in KD sera of the 1^st^ study period. One (m/z 1414.3) of the 5 serum KD-specific MAMPs (Figure 5A) and 2 other serum KD-specific MAMPs (m/z 745.6, 733.2) were detected in the IgG sepharose-binding fractions. The latter two were minor KD-specific MAMPs because they were detected in KD serum samples only after IgG sepharose purification. The MS/MS fragmentation patterns of the 3 molecules were similar to those of biofilm lipid extracts from *B.cereus*, while that of a molecule at m/z 733.2 also showed some similarity to that from *Y. pseudotuberculosis* (Figure S3 in File S1). To determine the IgG binding region of KD-specific MAMPs, polyclonal IgG, monoclonal IgG, F(ab′)_2_, and Fc affinity columns were employed. Serum KD-specific MAMPs bound to IgG mainly via Fab non antigen-binding regions (Figure 5B).

### Studies on the *in vitro* biofilm MAMPs from various microbes

**Figure 6:**
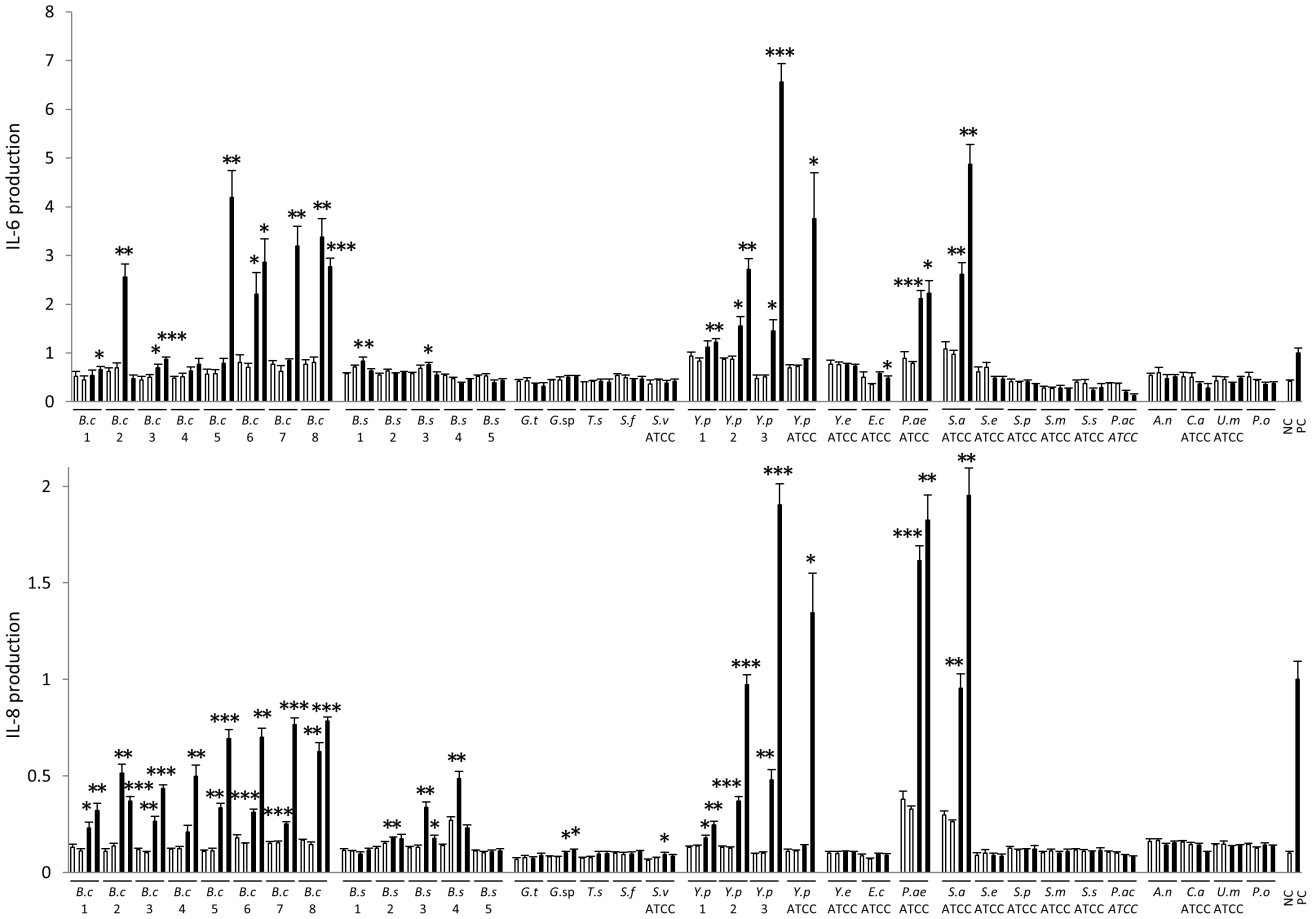
Activation of HCAECs by biofilm lipid extracts from various microbes. The production of IL-6 and IL-8 by HCAECs was measured after 24-h culture in the presence or absence of a microbial stimulant. Each microbe was cultured in the presence or absence of biofilm-forming glass slides with or without butter. As a microbial stimulant, an extract from a culture supernatant (□) or a biofilm (▪) of a microbe cultured in the presence (right column) or absence (left column) of butter was used. Medium alone, ethyl acetate alone or ethyl acetate extract from glass slides cultured in the absence of a microbe was used as a negative control (NC). FK 565 (10 µg/mL) was used as a positive control (PC). *B.c: Bacillus cereus, B.s: Bacillus subtilis, G.t: Gordonia terrae, T.s: Terribacillus saccharophilus, S.f: Streptomyces flavogriseus, S.v: Streptomyces violaceus, Y.p: Yersinia pseudotuberculosis, Y.e: Yersinia enterocolitica, E.c: Escherichia coli, P.ae: Pseudomonas aeruginosa, S.a: Staphylococcus aureus, S.e: Staphylococcus epidermidis, S.p: Streptococcus pyogenes, S.m: Streptococcus mitis, S.s: Streptococcus sanguinis, P.ac: Propionibacterium acnes, A.n: Aspergillus niger, C.a: Candida albicans, U.m: Ustilago maydis, P.o: Penicillium oxalicum*. Numbers under bacteria indicate those of KD patients. Data are expressed as the fold change induction of IL-8 or IL-6 compared to the PC levels. We performed the experiments 3 times. Biofilms were compared with supernatant control considering presence or absence of butter. *P<0.01, **P<0.001 and ***P<0.0001 (Welch's *t*-test).

**Figure 7:**
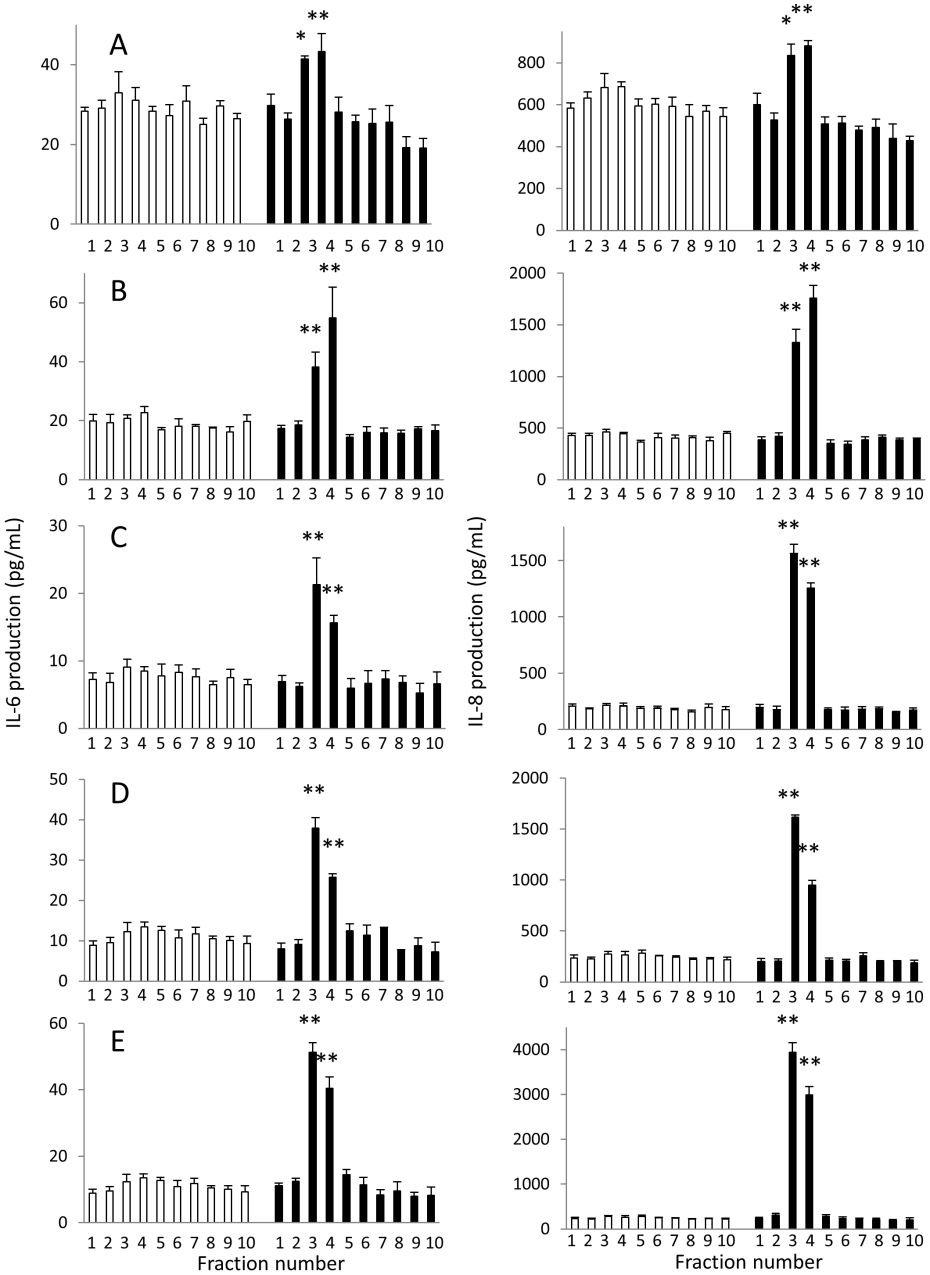
Fractionation of HCAEC-activating biofilm lipid extracts by HPLC. Each biofilm lipid extract from *Y. pseudotuberculosis* (A), *B. cereus* (B), *B. subtilis* (C), *S. aureus* (D) or *P. aeruginosa* (E) was separated into 10 fractions by HPLC and assayed for the stimulatory activity of HCAECs (▪). Fractions 3 and 4 from each biofilm lipid extract induced high cytokine production by HCAECs. Ethyl acetate lipid extracts from glass slides in the absence of microbes served as negative controls (□). Results are representative of 3 independent experiments. The stimulatory effects of fractionated biofilm samples were compared with those of corresponding controls. *P<0.05, **P<0.01 (Welch's *t*-test).

We investigated the stimulatory effects of extracts from culture supernatants or *in vitro* biofilms from various microbes on HCAECs. The biofilm extracts from *B. cereus* (9 out of 9 strains), *B. subtilis* (2 out of 5), *Y. pseudotuberculosis* (4 out of 4), *Pseudomonas (P.) aeruginosa* and *S. aureus* robustly induced the production of IL-8 and/or IL-6 by HCAECs, especially when microbes were cultured in the presence of sterilized butter (Figure 6). Biofilm extracts from *B. cereus*, *B. subtilis*, *Y. pseudotuberculosis*, *P. aeruginosa* and *S. aureus* were further fractionated by HPLC. In all of these 5 bacteria, HCAEC-stimulatory activity was observed in the same fractions (Figure 7). LC-MS analysis revealed that there were no common MAMPs in the fractions with high HCAEC-stimulatory activity among the biofilm extracts from *Y. pseudotuberculosis*, *B. cereus*, *B. subtilis*, *S. aureus* and *P. aeruginosa*.

## Discussion

The present study showed that serum KD-specific molecules had distinct m/z and MS/MS fragmentation patterns in each temporal clustering of outbreaks. These findings are consistent with the fact that cases in each cluster share similar clinical features [21].

At the 1^st^ study period, we detected 5 KD-specific molecules in patients' sera that were common to MAMPs from *in vitro* biofilms (4 from *B. cereus*, and 1 from *Y. pseudotuberculosis/S. aureus*). At the 2^nd^ and 3^rd^ study periods, we detected 4 and 3 serum KD-specific molecules in patients' sera, respectively, common to MAMPs from *in vivo* biofilms (1 from *B. cereus*, 1 from *B. subtilis*/*B. cereus*/*Y. pseudotuberculosis*, and 1 from *S. aureus*) in the respective KD patients. Although *Y. pseudotuberculosis* is sometimes involved in KD development [15], [16], the detection rate of *Y. pseudotuberculosis*-type MAMPs was low in our study. Rather, *B. cereus*-type MAMPs were most frequently associated with KD, and indeed *B. cereus* itself was isolated from our patients. In addition, microbes producing *B. subtilis*-type and *S. aureus*-type MAMPs were also associated with KD.

*B. cereus*, *B. subtilis*, *Y. pseudotuberculosis, S. aureus*, and *P. aeruginosa* produced endothelial cell-activating MAMPs only in the biofilm-forming conditions, mostly in higher amounts in the presence of butter (Figure 6). Four of the 5 bacteria were isolated from our KD patients, and *P. aeruginosa* was associated with KD development [22] and isolated from the small intestine of KD patients [23].

The biofilm formation may be found in living tissues including teeth, tongue, respiratory tract, middle ears, and gastrointestinal tract [24]. In KD, specific MAMPs were detected in sera as well as in the *in vivo* biofilm extracts from various sites by LC-MS analysis. Several molecules common to both KD patients' *in vivo* biofilms and sera were not present in the *in vitro* biofilm extracts of a single microbe, probably because they were products from polymicrobial biofilms *in vivo* [25]. The transition from the planktonic state to the sessile state in the biofilm induces a radical change in the gene and protein expression in bacteria. The biofilm matrix, composed of polysaccharides, proteins, nucleic acids and lipids, is newly produced and secreted to form the immediate extracellular environment [26]. Indeed, bacterial biofilm products were reported to induce a distinct inflammatory response in human cells compared to their planktonic counterparts [27]. In our study, not culture supernatants but biofilm extracts induced cytokine production in human endothelial cells (Figure 6).

*Bacillus* species including *B. cereus* and *B. subtilis* are volatile spore-forming rods widely distributed in soil and air, and sometimes induce infections and intoxications [28], [29]. The necessity of the biofilm and a certain environmental condition might explain why the presence of *Bacillus* species in control individuals does not induce KD by itself, and why other types of *Bacillus* species infections such as bacteremia and meningitis are not associated with KD development. In addition, just like KD [2], there is no person to person transmission in *Bacillus* species*-*associated human diseases such as food poisoning [28] and anthrax [30].

At least some serum KD-specific MAMPs bound to IgG mainly via Fab non antigen-binding regions, just like other microbial glycolipids that showed a high binding affinity to human IgG via Fab constant regions [19], [20]. Therefore, it is likely that high-dose IVIGs work, at least in part, as a scavenger of such MAMPs from the blood stream, a previously unrecognized mechanism in KD [31], [32].

The main limitations of our study were that the structural analysis of these KD-specific MAMPs was hampered by the instability of the lipophilic molecules after purification, and that fractionated crude biofilm extracts were too toxic to replicate the KD phenotype in mice.

We have shown that serum KD-specific molecules were diverse but mostly derived from biofilms and possessed molecular structures common to MAMPs. The present study suggests a possibility that KD-specific MAMPs induce vascular inflammation, leading to the development of KD. Further study is on the way as a nation-wide project to investigate a pathogenic link between KD development and biofilm-derived MAMPs.

## Conclusion

Extensive analysis by LC-MS/MS revealed that serum KD-specific molecules possessed molecular structures common to MAMPs from *Bacillus cereus, Bacillus subtilis*, *Yersinia pseudotuberculosis and Staphylococcus aureus*. These molecules were mostly derived from biofilms formed *in vivo* (teeth, tongue, nasal cavity, or stool). This report might offer novel insight into the diagnosis and management of KD as well as its pathogenesis.

## Supporting Information

File S1 **Supporting information.** Text S1, Supporting Materials and Methods. Figure S1, LC-MS chromatograms and the detection rates and time course of 5 KD-specific molecules. Figure S2, Effects of biofilm formation, shaking time and various oils on the production of a KD-specific MAMP by LC-MS analysis. Figure S3, LC-MS and MS/MS analyses of 3 KD-specific molecules with IgG sepharose-binding activity. Table S1, Detection rates of spore-forming and pathogenic microbes in the oral cavity and upper respiratory tract of KD patients. Table S2, Presence of MAMPs in various microbes similar to serum KD-specific molecules. Table S3, Common MAMPs between the *in vivo* biofilms and sera in respective KD patients at the 2^nd^ study. Table S4, Common MAMPs between the *in vivo* biofilms and sera, and microbes detected in respective KD patients at the 3^rd^ study. Table S5, Sequences of oligonucleotide primers used for the amplification microbial genes.
(PDF)
