# Supplementary material for: Kawasaki Disease-Specific Molecules in the Sera Are Linked to Microbe-Associated Molecular Patterns in the Biofilms
Source: PLoS One. 2014 Nov 20;9(11):e113054. doi: 10.1371/journal.pone.0113054 (PMC4239021; doi:10.1371/journal.pone.0113054)
Supplement: File S1 — Supporting information. Text S1, Supporting Materials and Methods. Figure S1, LC-MS chromatograms and the detection rates and time course of 5 KD-specific molecules. Figure S2, Effects of biofilm formation, shaking time and various oils on the production of a KD-specific MAMP by LC-MS analysis. Figure S3, LC-MS and MS/MS analyses of 3 KD-specific molecules with IgG sepharose-binding activity. Table S1, Detection rates of spore-forming and pathogenic microbes in the oral cavity and upper respiratory tract of KD patients. Table S2, Presence of MAMPs in various microbes similar to serum KD-specific molecules. Table S3, Common MAMPs between the in vivo biofilms and sera in respective KD patients at the 2nd study. Table S4, Common MAMPs between the in vivo biofilms and sera, and microbes detected in respective KD patients at the 3rd study. Table S5, Sequences of oligonucleotide primers used for the amplification microbial genes. (PDF) [file pone.0113054.s001.pdf]

Text S1. Supporting Materials and Methods

Luciferase Assay

NOD1-overexpressing HEK293 (NOD1 HEK293) cells were purchased from InvivoGen. Wild-type (WT) HEK293 cells were cultured in Dulbecco's modified Eagle's medium (DMEM) (Wako) supplemented with 10% fetal bovine serum (Invitrogen), 100 IU/ml penicillin and 100 μg/ml streptomycin. NOD1 HEK293 cells were cultured in DMEM supplemented with 10% fetal bovine serum, 50 IU/ml penicillin (Invitrogen), 50 μg/ml streptomycin (Invitrogen), 100 μg/ml Normocin™ (Invitrogen) and 10 μg/ml Blasticidin (InvivoGen). Transfection of plasmid DNA to HEK293 cells were performed using Effectene Transfection Reagent (QIAGEN) according to the manufacturer's protocol as described previously[1]. Seventy nanograms of pNF-κB-luc and 7 ng pRL-TK vectors were co-transfected. The pRL-TK vector contains the *Renilla luciferase* gene from *Renilla reniformis* transcribed by the TK promoter. The cells (2.6×10^4 per well) were plated in 96 well plates and after 4 hours incubation at 37°C in 5% CO_2, 3.75 μl of each sample and various reagents were added in the total of 75 μl medium. After 16 hours incubation at 37°C in 5% CO_2, luciferase activities were measured in triplicate by the GloMax®-Multi Detection System apparatus

(Promega) using the Dual-Glo Luciferase Assay System (Promega) according to the manufacturer's protocol. In all assays, relative luciferase activity was calculated as the ratio of firefly to *Renilla* luciferase activity. We performed these experiments 4 times. FK 565 (10 µg/mL) was used as a positive control in each experiment.

Authentic microbial glycolipids

Mannosylerythritol lipid, sophorolipid and cellobiose lipid were kindly provided by Kitamoto, D., National Institute of Advanced Industrial Science and Technology, Japan, and rhamnolipid was by Meitoh Kasei Co.,Ltd. Trehalose lipid was purchased from Carbosynth. These lipids were dissolved in ethyl acetate for analysis.

Microorganisms and culture

Bacillus cereus (*B. cereus*) (8 strains), *Bacillus subtilis* (*B. subtilis*) (5 strains), *Gordonia terrae* (*G. terrae*), *Terribacillus saccharophilus* (*T. saccharophilus*), *Streptomyces flavogriseus* (*S. flavogriseus*), *Aspergillus niger* (*A. niger*), and *Penicillium oxalicum* (*P. oxalicum*) were obtained from KD patients at Fukuoka Children's Hospital. *Yersinia pseudotuberculosis* (*Y. pseudotuberculosis*) (3 strains) was obtained from the stool of 3 KD patients at Fukuoka Children's

Hospital, Kawasaki Medical School Hospital, and Kurashiki Central Hospital. *Y. pseudotuberculosis* (1 strain), *Yersinia enterocolitica* (*Y. enterocolitica*), *Escherichia coli* (*E. coli*), *Pseudomonas aeruginosa* (*P. aeruginosa*), *Streptococcus pyogenes* (*S. pyogenes*), *Streptococcus sanguinis* (*S. sanguinis*), *Streptococcus mitis* (*S. mitis*), *Staphylococcus aureus* (*S. aureus*), *Staphylococcus epidermidis* (*S. epidermidis*), *Propionibacterium acnes* (*P. acnes*), *Streptomyces violaceus* (*S. violaceus*), *Candida albicans (C. albicans),* and *Ustilago maydis (U. maydis)* were purchased from American Type Culture Collection (ATCC).

Most spore-forming bacteria were cultured in Schaeffer's sporulation medium[2] (Difco sporulation medium (BD), containing, per liter, 8 g of Bacto-nutrient broth (Wako), 10 ml of 10% KCl, 10 ml of 1.2% $MgSO_4 \cdot 7H_2O$, 0.5 ml of 1 M NaOH, 1.0 ml of 1 M $Ca(NO_3)_2$, 1.0 ml of 0.01 M $MnCl_2$, 1.0 ml of 1 mM $FeSO_4$). After 48 hours incubation at 30°C, all colonies were suspended in 2 mL PBS (phosphate buffered saline) and heated at 80°C for 30 minutes to sterilize non spore-forming bacteria. Heated microbial suspensions were cultured in Trypto soy agar (Eiken) for 24 hours at 30 °C, and the single colonies were picked up and stored in 10% glycerol at -80 °C. *Streptomyces* species were cultured in yeast extract-malt

extract agar (BD) with 50 mg/dL of cycloheximide at 25 °C[3]. After 7-14 days, *Streptomyces* species were identified by the morphological characteristics[4] and 16s ribosomal RNA (rRNA) gene sequence[5, 6] (Table S5). Then, it was suspended in 15% glycerol and stored at -80 °C. Fungi were cultured in Sabouraud agar (Nissui) with 0.5 g/liter of gentamicin (SIGMA) and 0.04 g/liter of chloramphenicol (SIGMA)[7]. After 72 hours incubation at 25 °C, the single colonies were picked up and stored on Sabouraud agar slants at 25 °C. For the routine diagnostic culture of pathogenic bacteria, Sheep Blood Agar, Mannitol Salt Agar with Egg Yolk, Chocolate Agar and Deoxycholate Hydrogen sulfide Lactose (DHL) agar (Nissui Pharmaceutical Co. Ltd.) were used.

Biofilm studies

B. cereus and *B. subtilis* were cultured in Landy modified medium (per liter) containing 20 g of D-glucose, 5 g of glutamic acid, 1 g of yeast extract, 1g of K_2HPO_4, 0.5 g of $MgSO_4 \cdot 7H_2O$, 0.5 g of KCl, 1.6 mg of $CuSO_4$, 1.2 mg of $Fe_2(SO_4)_3$, 0.4 mg of $MnSO_4$, 2.2 g of $(NH_4)_2SO_4$ and 100 ml of 1 M MOPS (3-(N-morpholino) propane sulfonic acid) solution adjusted to pH 7.0 by 10 M KOH as described[8]. *Y. pseudotuberculosis, Y. enterocolitica, E. coli, P. aeruginosa, S. aureus, S. epidermidis, S. pyogenes, S. mitis,* and *S. sanguinis*

were cultured in BHI (Eiken). *Gordonia* sp., *G. terrae, T. saccharophilus, S. flavogriseus, S. violaceus,* and *P. acnes* were in Tryptic soy broth (Eiken). *C. albicans, U. maydis, A. niger and P. oxalicum* were in YM broth (BD). Butter (UNSALTED BUTTER, Snow Brand Milk Products Co., Ltd.) and horse oil (SON BAYU, Yakushido) were melted and mixed with DMSO. Olive oil (EXTRAVIRGIN OLIVE OIL BOSCO, The Nisshin OilliO Group, Ltd.), soybean oil (KENKOU SARARA, J-OIL MILLS , Inc.), sunflower oil (Olein Rich, Showa Sangyo Co., Ltd.) and cod oil (Norwegian Cod Liver Oil, Twinlab Corporation) were just mixed with DMSO. Butter or oil was filtered by 0.22 μm pore size cellulose acetate filter (Sartorius Stedim Biotech). To prepare seed cultures for biofilm studies, bacteria were cultured in a 50 mL conical tube with 15 mL of medium and two glass slides (76 × 26 × 1.2 mm, Matsunami)[9] with agitation for 72 hours at 30°C (*Gordonia* sp., *G. terrae, T. saccharophilus, S. flavogriseus,* or *S. violaceus*), for 24 hours at 37 °C (other bacteria) or for 96 hours at 25 °C (fungi). After the glass slides in the tube were washed twice with PBS, 15 mL of new medium with or without a various kind of oil/butter (3% v/v) was added. The optimal concentration of 3% v/v of oil/butter in DMSO was determined in preliminary experiments. Bacteria were further cultured with or without shake for additional 24, 48 or 72 hours at

the temperature of seed cultures.

Determination of microbes by PCR and sequencing

Total DNA was extracted by using the Illustra Bacteria GenomicPrep Mini Spin Kit (GE Healthcare) after boiling the suspension of microbes for 10 min. Subsequently, the DNA was subjected to PCR amplification of near-complete 16s rRNA genes using primers 8F and 1510R (Table S4), and gyrase B (*gyrB*) genes using primers UP1TL and UP2rTL (Table S5[5, 10, 11]). As for 16s *rRNA* genes, each 25 μL PCR reaction mixture contained 1 μL DNA extract, 0.2 mM of each dNTP, 0.4 μM of each primer, and 1.5 U rTaq DNA polymerase with 1x PCR buffer. A total of 30 cycles of amplification was performed with denaturation at 96 °C for 1 minute, annealing at 55 °C for 1 minute, and an extension at 72°C for 1 minute. As for *gyrB* genes, each 50 μL PCR reaction mixture contained 1 μL DNA extract, 0.2 mM of each dNTP, 0.2 μM of each primer, and 1.5 U rTaq DNA polymerase with 1x PCR buffer. Amplification of *gyrB* genes was performed as follows: an initial denaturation at 94 °C for 3 minutes, 30 cycles of amplification with denaturation at 94 °C for 30 seconds, annealing at 50 °C for 60 seconds and an extension at 72°C for 90 seconds, and a final extension at 72 °C for 5 minutes. As for fungi, the DNA was subjected to amplification of the ITS-1

region fragment. The primers used for the amplification of the ITS-1 region were ITS1F and ITS1R[12] (Table S5). Each 50 μL PCR reaction mixture contained 1 μL DNA extract, 0.2 mM of each dNTP, 0.2 μM of each primer, and 1.5 U rTaq DNA polymerase with 1x PCR buffer. A total of 35 cycles of amplification was performed with denaturation at 94 °C for 30 seconds, annealing at 55 °C for 1 minute, and an extension at 72°C for 1 minute. The products were analyzed by electrophoresis on a 1% agarose gel stained with ethidium bromide. The PCR product was purified with the Wizard® SV Gel and PCR Clean-Up System (Promega). The 16s *rRNA*, *gyrB* and the *ITS-1* gene sequences were obtained using the BigDye Terminator v3.1 Cycle Sequencing Kit (Applied Biosystems) and the ABI PRISM 3100 or 3130 Genetic Analyzer (Applied Biosystems). DNA was partially sequenced using primers 8F, 519F, 907F, 342R, UP1TL, UP-2rTL, ITS1F and ITS1R (Table S4), and the sequences were assembled from the combination of separate fragments generated with forward and reverse sequencing primers using the BioEdit Sequence Alignment Editor. The sequences were identified using the basic local alignment search tool (BLAST).

B. cereus group (*B. cereus*, *B. thuringiensis*, *B. anthracis* and *B. mycoidis*) and *B. subtilis* group (*B. subtilis*, *B. pumilus*, *B. atrophaeus*, *B. licheniformis* and *B.*

amyloliquefaciens) were also determined by specific PCR. Collected DNA samples were subjected to PCR amplification of *groEL* genes using primers BCGHS-1F and BCGSH-1R to detect *B. cereus* group[13], and 16s rRNA genes using primers Bsub5F and Bsub3R to detect *B. subtilis* group[14] (Table S5). Each 25 µL PCR reaction mixture contained 1 µL DNA extract, 0.2 mM of each dNTP, 0.4 µM of each primer, and 1.5 U rTaq DNA polymerase with 1x PCR buffer. The reaction parameters were an initial denaturation at 94 °C for 5 minutes, 30 cycles of amplification with denaturation at 94 °C for 30 seconds, annealing at 63°C for 30 seconds, an extension at 72 °C for 30 seconds, and a final extension at 72 °C for 5 minutes. The amplified products were analyzed by electrophoresis on 1% agarose gels. The 16s rRNA genes of *B. cereus group* bacteria are quite similar[15]. The DNA samples that were classified to be *B. cereus* group by 16s rRNA sequence were further subjected to PCR amplification of gyrase B subunit gene (*gyrB*)[11] to determine a *Bacillus* species within *B. cereus* group (Table S5). Each 25 µL PCR reaction mixture contained 1 µL DNA extract, 0.2 mM of each dNTP, 0.4 µM of each primer, and 1.5 U rTaq DNA polymerase with 1x PCR buffer. The reaction parameters were an initial denaturation at 94 °C for 5 minutes, 30 cycles of amplification with denaturation

at 94 °C for 30 seconds, annealing at 63°C for 30 seconds, an extension at 72°C for 30 seconds, and a final extension at 72 °C for 5 minutes. The amplified products were analyzed by electrophoresis. To confirm that the *B. anthracis*-like strain, which showed a *gyrB* gene sequence very similar to that of *B. anthracis,* was really *B. anthracis*, we first performed PCR amplification of protective antigen (PA) and capsule genes that are specific for *B. anthracis*[16]. Each 25 μL of PCR reaction mixture contained 1 μL DNA extract, 0.2 mM of each dNTP, 0.4 μM of each primer, and 1.5 U rTaq DNA polymerase with 1x PCR buffer. The reaction parameters were an initial denaturation at 94 °C for 5 minutes, 30 cycles of amplification with denaturation at 94 °C for 30 seconds, annealing at 50 °C for 30 seconds and an extension at 74°C for 60 seconds. The amplified products were analyzed by electrophoresis. Second, we examined bacteriological characteristics (motility and hemolysis) of *B. anthracis*-like strains[17].

Fractionation of bacterial biofilm extracts by HPLC

Biofilm extracts were fractionated into 10 fractions by HPLC (Agilent 1200 HPLC instrument, Agilent Technologies) on Dionex Acclaim surfactant column (3 μm, 120 Å, 2.1 × 150 mm, DIONEX). The mobile phases were H_2O with 0.1% formic acid (eluent A) and acetonitril with 0.1% formic acid (eluent B). They were

delivered at a flow rate of 0.2 ml/min and the column was operated at 25 °C. The gradient was as follows: 0-3 min. 20% B, 3-12 min. 20-100% B, 12-70 min. 100% B. The injection volume was 900 µL. All 10 fractions were evaporated and the pellets were suspended in 100% methanol and assayed for HCAEC-activating activity.

Supporting References:

1. Makimura M, Ihara K, Kojima-Ishii K, Nozaki T, Ohkubo K, et al. (2011) The signal transducer and activator of transcription 5B gene polymorphism contributes to the cholesterol metabolism in Japanese children with growth hormone deficiency. Clin Endocrinol (Oxf) 2011 74: 611-617.
2. Nicholson WL, Setlow P. (1990) Sporulation, germination and outgrowth. In: Harwood CR and Cutting SM, editors. Molecular biological methods for *Bacillus*. New York: John Wiley & Sons, Inc. pp. 1990:391–450.
3. Cuesta G, García-de-la-Fuente R, Abad M., Fornes F. (2012) Isolation and identification of actinomycetes from a compost-amended soil with potential as biocontrol agents. J Environ Manage 95: S280-284.
4. Salamoni SP, Mann MB, Campos FS, Franco AC, Germani JC, Sand VD.

(2010) Preliminary characterization of some Streptomyces species isolated from a composting process and their antimicrobial potential. World J Microbiol Biotechnol 26: 1847-1856.

5. Lane DJ (1991) 16S/23S rRNA sequencing. In: Stackebrandt E, Goodfellow M, editors. Nucleic acid techniques in bacterial systematics. New York: John Wiley & Sons, Inc. pp. 115-175.

6. Vescio PA, Nierzwicki-Bauer SA. (1995) Extraction and purification of PCR amplifiable DNA from lacustrine subsurface sediments. J Microbiol Methods 21: 225-233.

7. Hauser PM, Bernard T, Greub G, Jaton K, Pagni M. (2014) Hafen GM: Microbiota present in cystic fibrosis lungs as revealed by whole genome sequencing. PLoS One 9: e90934.

8. Shakeri Fard P (2010) Production and purification of biosurfactants and study of their influence on surface properties of stainless steel and Teflon. University of Lille 1, Science and Technology. Available: http://ori.univ-lille1.fr/notice/view/univ-lille1-ori-14810. Accessed 30 December 2011.

9. Wu MC, Lin TL, Hsieh PF, Yang HC, Wang JT. (2011) Isolation of genes

involved in biofilm formation of a Klebsiella pneumoniae strain causing pyogenic liver abscess. PLoS One 6: e23500.

10. Vescio PA, Nierzwicki-Bauer SA. (1995) Extraction and purification of PCR amplifiable DNA from lacustrine subsurface sediments. J Microbiol Methods 21: 225-233.

11. Kasai H, Ezaki T, Hirayama S. (2000) Differentiation of phylogenetically related slowly growing mycobacteria by their gyrB sequences. J Clin Microbiol 38:301-308.

12. Abe M, Takaoka N, Idemoto Y, Takagi C, Imai T. (2008) Characteristic fungi observed in the fermentation process for Puer tea. Int J Food Microbiol 31:199-203.

13. Park SH, Kim HJ, Kim JH, Kin TW, Kim HY. (2007) Simultaneous detection and identification of Bacillus cereus group bacteria using multiplex PCR. J Microbiol Biotechnol 17:1177-1182.

14. Wattiau P, Renard ME, Ledent P, Debois V, Blackman G (2001) Agathos SN: A PCR test to identify Bacillus subtilis and closely related species and its application to the monitoring of wastewater biotreatment. Appl Microbiol Biotechnol 56:816-819.

15. Radnedge L, Agron PG, Hill KK, Jackson PJ, Ticknor LO. (2003) Andersen GL: Genome differences that distinguish Bacillus anthracis from Bacillus cereus and Bacillus thuringiensis. Appl Environ Microbiol 69:2755-2764.

16. World Heath Organization (WHO). Guidelines for the surveillance and control of anthrax in humans and animals. Third ed., 1998. WHO/EMC/ZDI./98.6.

17. Centers for Disease Control and Prevention (CDC), American Society for Microbiology (ASM), and Association of Public Health Laboratories (APHL). Basic diagnostic testing protocols for level A laboratories for the presumptive identification of Bacillus anthracis. 2002. ban.asm.la.cp.031802.

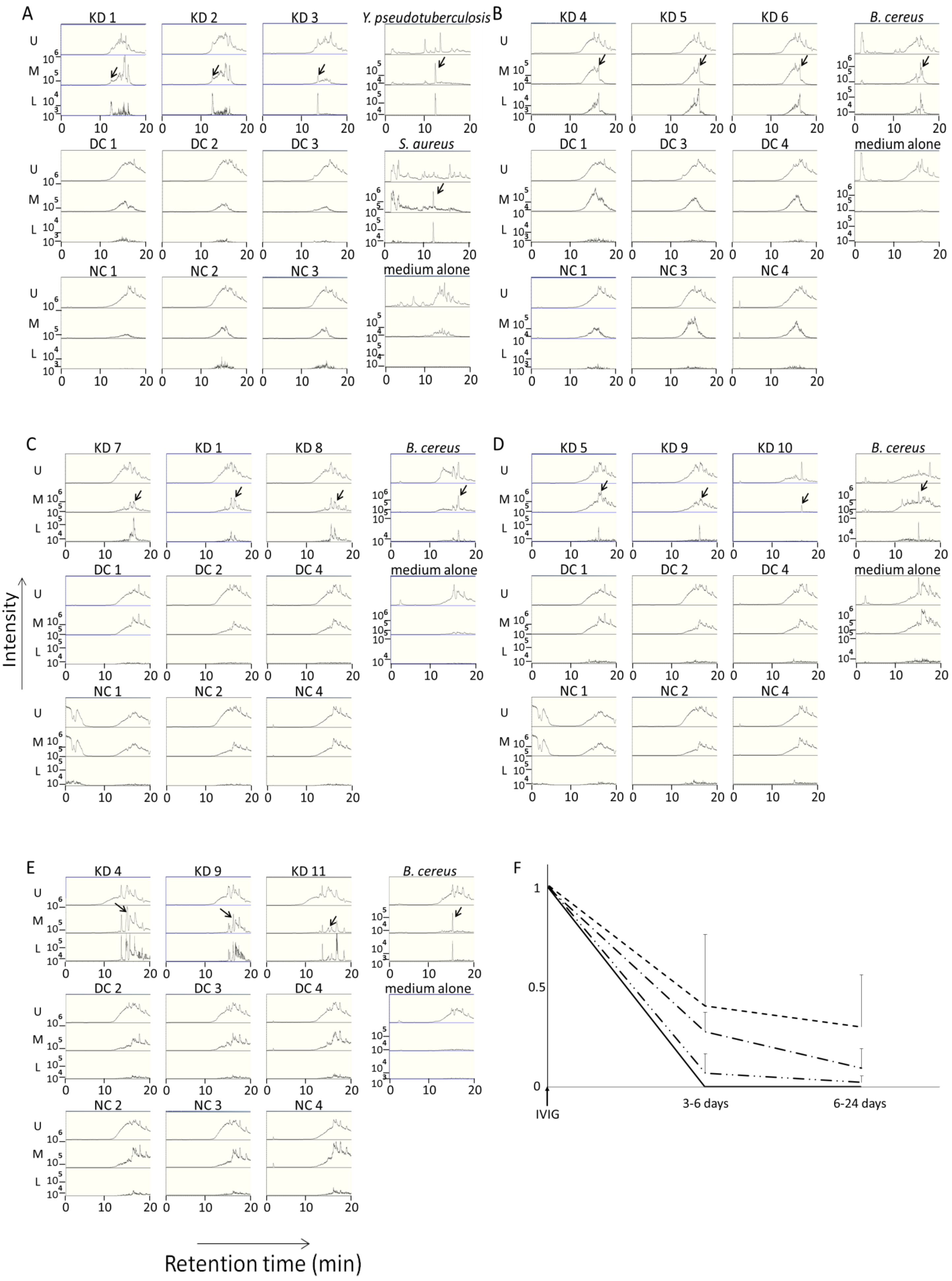
A
KD 1
KD 2
KD 3
Y. pseudotuberculosis
DC 1
DC 2
DC 3
S. aureus
NC 1
NC 2
NC 3
medium alone
B
KD 4
KD 5
KD 6
B. cereus
DC 1
DC 3
DC 4
medium alone
NC 1
NC 3
NC 4
C
KD 7
KD 1
KD 8
B. cereus
DC 1
DC 2
DC 4
medium alone
NC 1
NC 2
NC 4
D
KD 5
KD 9
KD 10
B. cereus
DC 1
DC 2
DC 4
medium alone
NC 1
NC 2
NC 4
E
KD 4
KD 9
KD 11
B. cereus
DC 2
DC 3
DC 4
medium alone
NC 2
NC 3
NC 4
U
M
L
Intensity
Retention time (min)
F
1
0.5
0
IVIG
3-6 days
6-24 days

Figure S1. LC-MS chromatograms and the detection rates and time course of 5 KD-specific molecules

LC-MS chromatograms of KD specific molecules (A: m/z 1531.8, B: m/z 1414.3, C: m/z 790.9, D: m/z 779.8, and E: m/z 695.0) are shown in 3 KD patients, 3 DC controls and 3 NC subjects (3 x 3 left panels). LC-MS chromatograms of biofilm lipid extracts from *Y. pseudotuberculosis* and *S. aureus* (A) and *B. cereus* (B-E) are also shown in the right panels. U: Total ion current chromatograms, M: Extracted-ion chromatograms at m/z 1500-1600 (A), m/z 1400-1500 (B), m/z 700-800 (C and D), and m/z 600-700 (E), L: Extracted-ion chromatograms at m/z 1531.8 (A), m/z 1414.3 (B), m/z 790.9 (C), m/z 779.8 (D), and m/z 695.0 (E). Arrows indicate peaks of target molecules. F: The intensities of all molecules decreased after high-dose intravenous immunoglobulin (IVIG) treatment. m/z 1531.8 and m/z 1414.3: solid line, m/z 790.9: dash line, m/z 779.8: one-dot chain line, m/z 695.0: two-dot chain line.

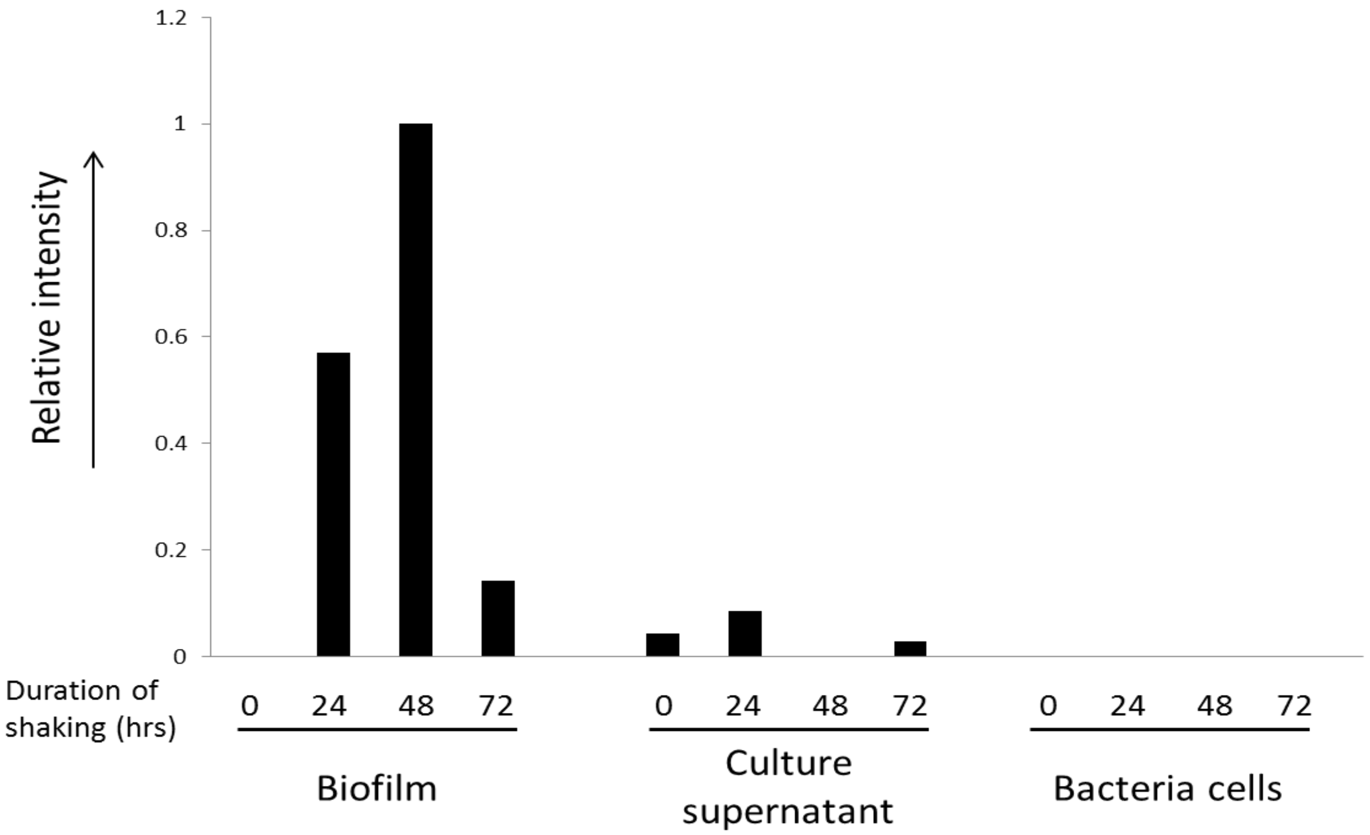

Relative intensity
1.2
1
0.8
0.6
0.4
0.2
0
Duration of shaking (hrs)
0 24 48 72
Biofilm
0 24 48 72
Culture supernatant
0 24 48 72
Bacteria cells

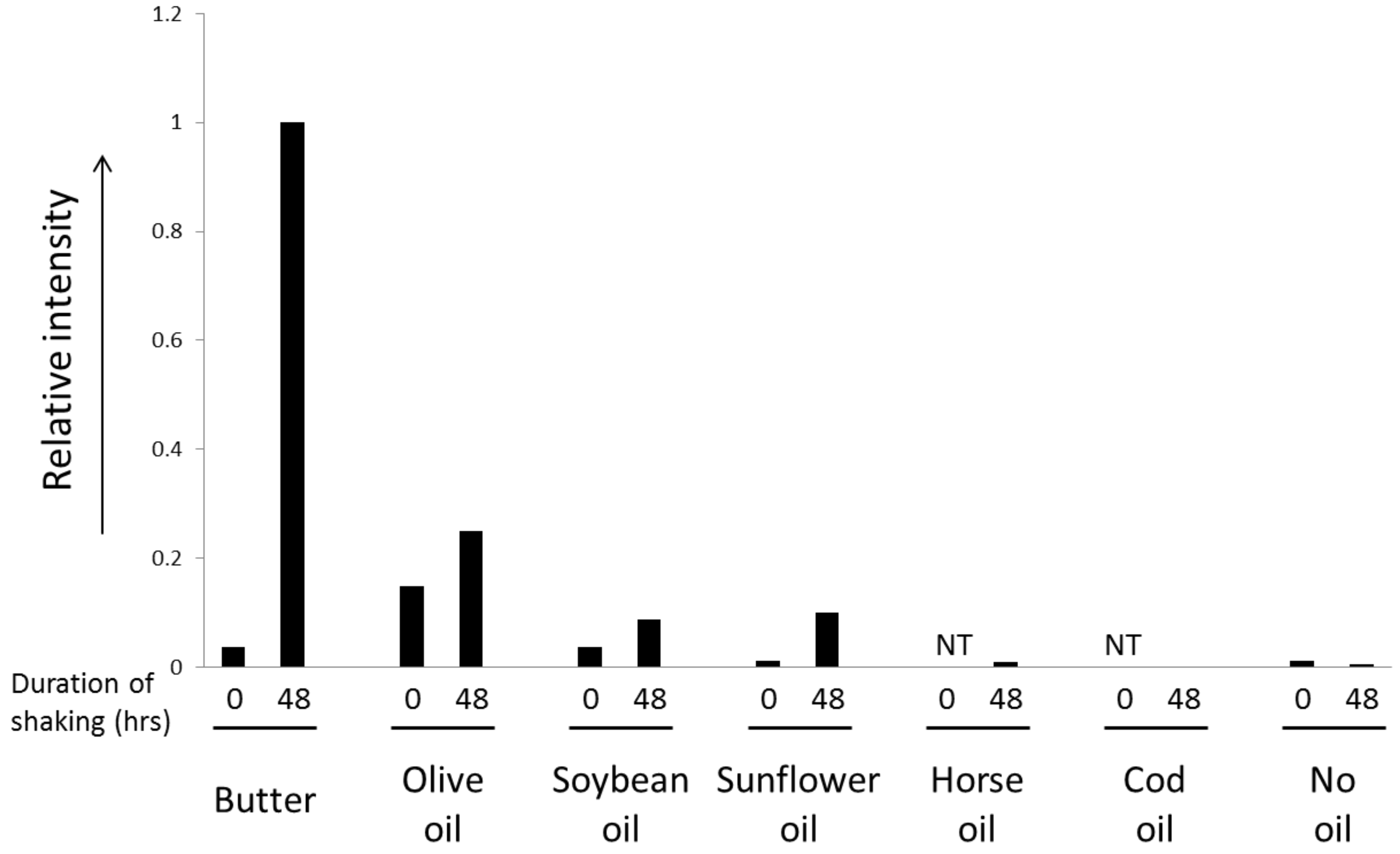

Relative intensity
1.2
1
0.8
0.6
0.4
0.2
0
NT
NT
Duration of shaking (hrs)
0 48
Butter
0 48
Olive oil
0 48
Soybean oil
0 48
Sunflower oil
0 48
Horse oil
0 48
Cod oil
0 48
No oil

Figure S2. Effects of biofilm formation, shaking time and various oils on the production of a KD-specific MAMP by LC-MS analysis

A. As for the seed on biofilm, bacteria (*B. cereus*) were cultured with agitation for 24 hours at 37 °C in 50 mL conical tube with 10 mL of medium and a glass slide. After wash with PBS twice, 10 mL of new medium was added to a conical tube with a glass slide and bacteria were further cultured for 72 hours with initial 0, 24, 48 or 72 hours of shake. The production of one (m/z 790.9) of the KD-specific molecules in lipid extracts from biofilms, culture supernatants or bacterial cells was determined by LC-MS analysis.

B. After the seed of bacteria on biofilm as described above, bacteria were further cultured for 72 hours with initial 0 or 48 hours of shake. Representative data are shown on the production of one of the KD-specific MAMPs in the presence or absence of butter in *DMSO*, olive oil, soybean oil, sunflower oil, horse oil in *DMSO*, or cod oil under the biofilm-forming condition. NT; not tested. The final DMSO concentration was 3%.

We performed the experiments at least 3 times and show one representative data.

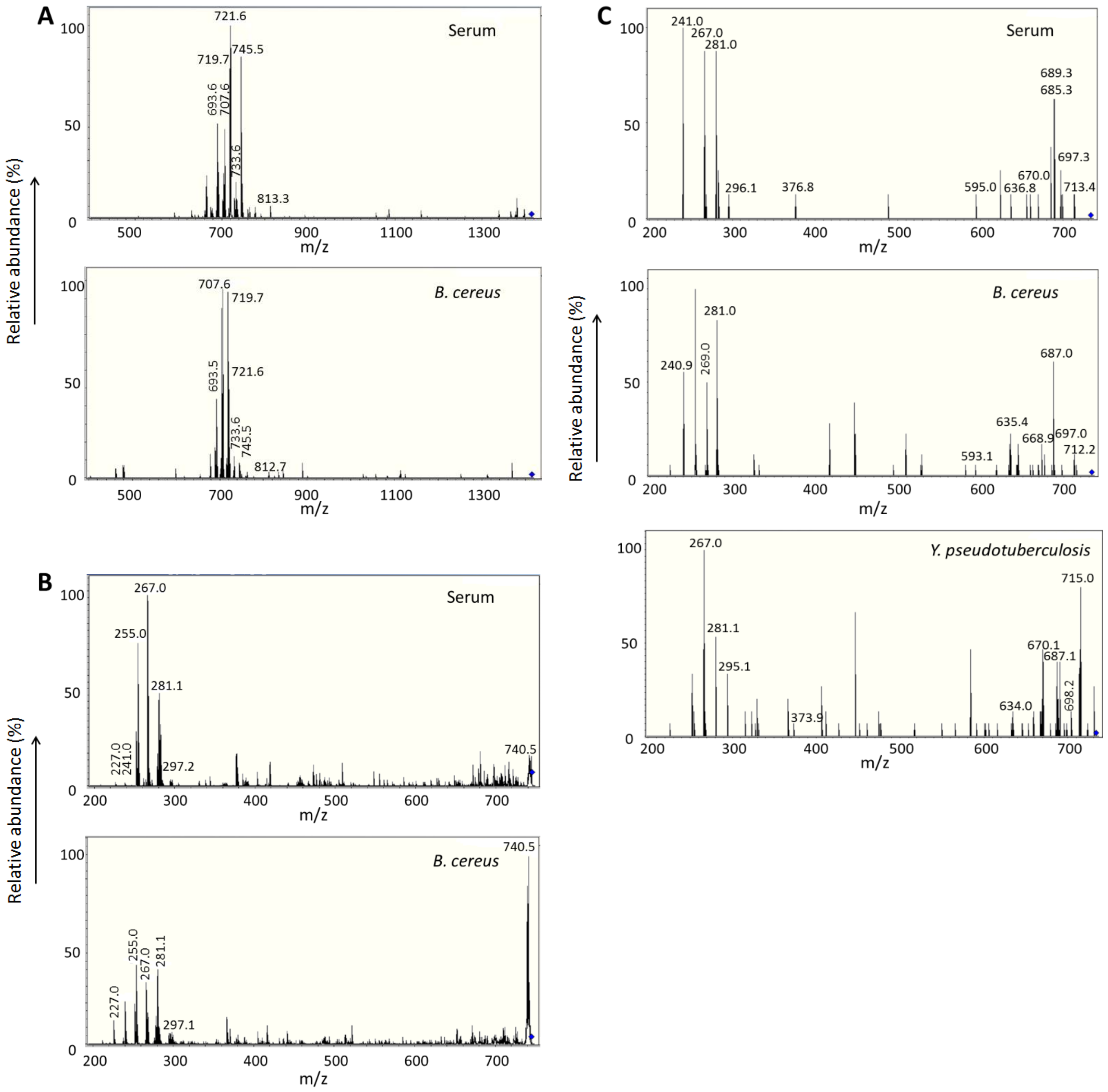

A
Serum
721.6
719.7
745.5
693.6
707.6
733.6
813.3
B. cereus
707.6
719.7
693.5
721.6
733.6
745.5
812.7
Relative abundance (%)
m/z
B
Serum
267.0
255.0
281.1
227.0
241.0
297.2
740.5
B. cereus
740.5
255.0
267.0
281.1
227.0
297.1
C
Serum
241.0
267.0
281.0
689.3
685.3
697.3
670.0
296.1
376.8
595.0
636.8
713.4
B. cereus
281.0
240.9
269.0
687.0
635.4
668.9
697.0
593.1
712.2
Y. pseudotuberculosis
267.0
715.0
281.1
295.1
670.1
687.1
698.2
634.0
373.9

Figure S3. LC-MS and MS/MS analyses of 3 KD-specific molecules with IgG sepharose-binding activity

Three KD-specific IgG sepharose-binding molecules were detected by extensive LC-MS analysis of KD sera with DC control sera and inactivated CNBr Sepharose as controls. By MS/MS analysis, IgG sepharose-binding KD-specific molecules (A. m/z 1414.3, B. m/z 745.6, C. m/z 733.2) and MAMPs from *B. cereus* show similar MS/MS fragmentation patterns. The MS/MS fragmentation pattern of a molecule at m/z 733.2 also shows some similarity to that of biofilm lipid extracts from *Y. pseudotuberculosis*.

Table S1. Detection rates of spore-forming and pathogenic microbes in the oral cavity and upper respiratory tract of KD patients

Isolate	KD patients *n*=27	DC controls *n*=19	*P*-value*
	No. (%)	No. (%)	
Spore-forming microbes**			
Bacillus cereus	8 (29.6)	1 (5.2)	0.06
Bacillus subtilis	4 (14.8)	0	0.13
Bacillus species	0	1† (5.2)	0.41
Streptomyces	1‡ (3.7)	0	1.00
Other bacteria	3§ (11.1)	1¶ (5.2)	0.63
Fungi susp.	0	2 (10.5)	0.17
Pathogenic bacteria			
Staphylococcus aureus	8 (29.6)	7 (36.8)	0.75
Streptococcus pyogenes	0	0	-
Pseudomonas aeruginosa	0	0	-

* two sided Fisher's exact test
** Spore-forming bacteria were obtained after heat treatment at 80°C for 30 minutes to sterilize non spore-forming bacteria.
† *Bacillus megaterium,* ‡ *Streptomyces flavogriseus,* § *Gordonia* species, *Gordonia terrae, Terribacillus shanxiensis/ saccharophilus,* ¶ *Rhodococcus kroppenstedtii/ corynebacterioides*
Some *B. cereus* strains were judged to be *B. anthracis*-like strains by the sequences of the *gyrB* gene. However, by the absence of protective antigen (PA) and capsule genes that are specific for *B. anthracis* and the presence of motility and beta-hemolysis on blood agar, we concluded that the strains were *B. cereus*.

Table S2. Presence of MAMPs in various microbes similar to serum KD-specific molecules

	1st study					2nd study				3rd study		
m/z (RT)	1531.8 (11.1)	1414.3 (15.7)	790.9 (16.0)	779.8 (15.8)	695.0 (14.9)	1171.4 (18.8)	1169.4 (18.6)	906.8 (19.8)	695.0 (14.9)	667.4 (15.4)	619.4 (15.6)	409.3 (17.4)
B. cereus	-	+	+	+	+	-	-	-	+	-	++	-
B. subtilis	-	-	-	-	-	-	-	-	-	-	++	-
G. terrae	+	-	-	-	-	-	-	-	-	-	-	-
T. saccharophilus	-	-	-	-	-	-	-	-	-	-	-	-
S. flavogriseus	-	-	-	-	-	-	-	-	-	-	-	-
S. violaceus	+	-	-	-	-	-	-	-	-	-	-	-
Y. pseudotuberculosis	++	-	-	-	-	-	-	-	-	-	++	-
Y. enterocolitica	+	-	-	-	-	-	-	-	-	-	-	-
E. coli	-	-	-	-	-	-	-	-	-	-	-	-
P. aeruginosa	-	-	-	-	-	-	-	-	-	-	-	-
S. aureus	++	-	-	-	-	-	-	-	-	++	-	-
S. epidermidis	+	-	-	-	-	-	-	-	-	-	-	-
S. pyogenes	++	-	-	-	-	-	-	-	-	-	-	-
S. mitis	-	-	-	-	-	-	-	-	-	-	-	-
S. sanguis	-	-	-	-	-	-	-	-	-	-	-	-
P. acnes	-	-	-	-	-	-	-	-	-	-	-	-
A. niger	-	-	-	-	-	-	-	-	-	-	-	-
C. albicans	-	-	-	-	-	-	-	-	-	-	-	-
U. maydis	-	-	-	-	-	-	-	-	-	-	-	-
P. oxalicum	-	-	-	-	-	-	-	-	-	-	-	-

Lipid extracts from biofilms of various microbes were analyzed for molecules with MS/MS fragmentation patterns similar to those of serum KD-specific molecules by LC-MS and MS/MS analyses.

+ Intensity < $1X10^5$ ++ Intensity ≥ $1X10^5$

Table S3. Common MAMPs between the *in vivo* biofilms and sera in respective KD patients at the 2nd study

	**a**					**b**					**c**					**d**				
m/z (RT)	1171.4 (18.8)					1169.4 (18.6)					906.8 (19.8)					695.0 (14.9)				
	Se	Te	To	N	St	Se	Te	To	N	St	Se	Te	To	N	St	Se	Te	To	N	St
S2-KD1	-	ND	-	ND	ND	-	ND	-	ND	ND	-	ND	-	ND	ND	+	ND	+	ND	ND
S2-KD2	-	ND	-	ND	ND	-	ND	-	ND	ND	-	ND	-	ND	ND	+	ND	+	ND	ND
S2-KD3	+	-	-	-	-	+	-	-	-	+	-	-	-	-	-	+	-	-	-	-
S2-KD4	+	+	+	+	-	+	-	-	-	-	-	-	-	-	-	+	-	-	-	-
S2-KD5	-	-	-	-	-	-	-	-	-	-	-	-	-	-	-	+	-	-	-	-
S2-KD6	-	-	-	-	-	-	-	-	-	-	-	-	-	-	-	-	-	-	-	-
S2-KD7	-	-	-	-	-	-	-	-	-	-	-	-	-	-	-	+	-	-	-	-
S2-KD8	-	-	-	-	-	-	-	-	-	-	+	+	-	-	-	-	-	-	-	-
S2-KD9	-	-	-	-	-	-	-	-	-	-	+	+	-	-	-	-	-	-	-	-
S2-KD10	-	-	-	-	-	-	-	-	-	-	+	+	-	-	-	-	-	-	-	-
S2-KD11	-	-	-	-	-	-	-	-	-	-	-	+	-	-	-	-	-	-	-	-
S2-KD12	-	-	-	-	-	-	-	-	-	-	+	-	-	-	-	-	-	-	-	-
S2-DC1	-	-	-	-	-	-	-	-	-	-	-	-	-	-	-	-	-	-	-	-
S2-DC2	-	-	-	-	-	-	-	-	-	-	-	-	-	-	-	-	-	-	-	-
S2-DC3	-	-	-	-	-	-	-	-	-	-	-	-	-	-	-	-	-	-	-	-
S2-DC4	-	-	-	-	-	-	-	-	-	-	-	-	-	-	-	-	-	-	-	-
S2-DC5	-	-	-	-	-	-	-	-	-	-	-	-	-	-	-	-	-	-	-	-
S2-DC6	-	-	-	-	-	-	-	-	-	-	-	-	-	-	-	-	-	-	-	-
S2-DC7	-	-	-	-	-	-	-	-	-	-	-	-	-	-	-	-	-	-	-	-
S2-DC8	-	-	-	-	-	-	-	-	-	-	-	-	-	-	-	-	-	-	-	-
S2-DC9	-	-	-	-	-	-	-	-	-	-	-	-	-	-	-	-	-	-	-	-
S2-DC10	-	-	-	-	-	-	-	-	-	-	-	-	-	-	-	-	-	-	-	-

KD: Kawasaki disease, DC: other febrile illnesses, Se: serum, biofilm extracts from teeth (Te), tongue (To), nose (N) and stool (St). ND: not determined

Table S4. Common MAMPs between the *in vivo* biofilms and sera, and microbes detected in respective KD patients at the 3rd study

	a			b			c								
m/z (RT)	667.4 (15.4)			619.4 (15.6)			409.3 (17.4)			*B. cereus*	*B. subtilis*	*S. aureus*	*S. pyogenes*	*P. aeruginosa*	*Y. pseudo-tuberculosis*
	Se	Te	To	Se	Te	To	Se	Te	To						
S3-KD1	+	-	+	-	+	+	+	+	+	-	+	+	-	-	-
S3-KD2	-	-	+	+	+	+	+	+	+	-	+	+	-	-	-
S3-KD3	+	-	+	-	+	+	+	+	+	-	+	-	-	-	-
S3-KD4	+	-	+	+	+	+	+	+	+	-	+	+	-	-	-
S3-KD5	-	-	+	+	+	+	+	+	+	-	+	-	-	-	-
S3-KD6	+	-	+	+	+	+	+	+	+	-	-	+	-	-	-
S3-KD7	-	-	+	+	+	+	+	+	+	-	+	-	-	-	-
S3-KD8	+	-	+	-	+	+	+	+	+	-	-	-	-	-	-
S3-KD9	+	-	+	-	+	+	+	+	+	-	+	-	-	-	-
S3-KD10	-	-	+	-	+	+	-	+	+	-	-	-	-	-	-
S3-KD11	+	-	+	-	+	+	-	+	+	-	+	-	-	-	-

KD: Kawasaki disease, Se: serum, Biofilm extracts from teeth (Te) and tongue (To)

B. cereus, *B. subtilis*, *S. aureus*, *S. pyogenes* and *P. aeruginosa* from oral or nasal cavity were determined by PCR (*B. cereus*, *B. subtilis*) or routine cultures (*S. aureus*, *S. pyogenes* and *P. aeruginosa*). Stool was used for routine culture of *Y. pseudotuberculosis*.

Table S5. Sequences of oligonucleotide primers used for the amplification microbial genes

16S *rRNA*, *gyrB* and *ITS-1* genes

Primer	Sequence (5' to 3')	Target gene
8F	AGA GTT TGA TCM TGG CTC AG	16S *rRNA*
342R	CTG CTG CSY CCC GTA G	16S *rRNA*
519F	CAG CMG CCG CGG TAA TWC	16S *rRNA*
907F	AAA CTY AAA KGA ATT GAC GG	16S *rRNA*
1510R	TAC GGY TAC CTT GTT ACG ACT T	16S *rRNA*
UP1TL	CAY GCN GGN GGN AAR TTY GA	*gyrB*
UP2rTL	TCN ACR TCN GCR TCN GTC AT	*gyrB*
ITS1F	GTA ACA AGG T(T/C)T CCG T	*ITS-1*
ITS1R	CGT TCT TCA TCG ATG	*ITS-1*

Bacillus-specific PCR

Species	Primer	Sequence (5' to 3')	Target gene
B. cereus group	BCGSH-1F	GTG CGA ACC CAA TGG GTC TTC	*groEL*
	BCGSH-1R	CCT TGT TGT ACC ACT TGC TC	*groEL*
B. cereus	BCJH-F	TCA TGA AGA GCC TGT GTA CG	*gyrB*
	BCJH-1R	CGA CGT GTC AAT TCA CGC GC	*gyrB*
B. anthracis	BASH-2F	GGT AGA TTA GCA GAT TGC TCT TCA AAA GA	*gyrB*
	BASH-2R	ACG AGC TTT CTC AAT ATC AAA ATC TCC GC	*gyrB*
B. thuringiensis	BTJH-1F	GCT TAC CAG GGA AAT TGG CAG	*gyrB*
	BTJH-R	ATC AAC GTC GGC GTC GG	*gyrB*
B. mycoides	BMSH-F	TTT TAA GAC TGC TCT AAC ACG TGT AAT	*gyrB*
	BMSH-R	TTC AAT AGC AAA ATC CCC ACC AAT	*gyrB*
B. subtilis group	Bsub5F	AAGTCGAGCGGACAGATGG	16s *rRNA*
	Bsub3R	CCAGTTTCCAATGACCCTCCCC	16s *rRNA*

B. anthracis-specific PCR

Primer	Sequence (5' to 3')	Target gene
PA5	TCC TAA CAC TAA CGA AGT CG	*PA*
PA8	GAG GTA GAA GGA TAT ACG GT	*PA*
1234	CTG AGC CAT TAA TCG ATA TG	*Capsule*
1301	TCC CAC TTA CGT AAT CTG AG	*Capsule*
